# Influence of Specific Acoustic Parameters on Responses in Growing Pigs: Towards a Precision Auditory Enrichment Strategy

**DOI:** 10.3390/ani16101475

**Published:** 2026-05-11

**Authors:** Zhijiang Wang, Mengyao Yi, Haoyuan Liu, Zhouhao Zhang, Haikang Li, Guangying Hu, Zhenyu Liu

**Affiliations:** 1 College of Information Science and Engineering, Shanxi Agricultural University, Taigu 030801, China; wangzhijiang910@163.com (Z.W.); 15234750515@163.com (M.Y.); 18734579029@163.com (H.L.); 16635583985@163.com (Z.Z.); 2 College of Agricultural Engineering, Shanxi Agricultural University, Taigu 030801, China; lhkang1993@126.com; 3 College of Animal Science, Shanxi Agricultural University, Taigu 030801, China; 4 Dryland Farm Machinery Key Technology and Equipment Key Laboratory of Shanxi Province, Taigu 030801, China

**Keywords:** animal welfare, auditory enrichment, acoustic parameter preference, precision analysis, automated computer vision

## Abstract

Improving animal welfare through standardized management protocols remains a key challenge in intensive pig production. Auditory enrichment, such as music, represents a promising non-invasive strategy, yet its application is often empirical, lacking mechanistic understanding and objective assessment tools. This study investigated growing pigs’ active preferences for structured musical parameters to establish a precision auditory enrichment framework. Seventy-two crossbred pigs were subjected to a free-choice paradigm under simulated farm conditions, with a 2 × 2 factorial design manipulating musical stimulus type (a guqin string piece vs. a Mozart wind excerpt) and tempo (fast: 200 bpm vs. slow: 65 bpm) was continuously quantified using an enhanced YOLOv11-based automated recognition system (mean average precision mAP_50_: 90.5% ± 1.5%). Results revealed highly parameter-dependent effects: the slow-tempo GS stimulus and the fast-tempo MF stimulus significantly prolonged occupancy time (*p* < *0.01*) and elicited distinct profiles. The GS stimulus promoted a calm, investigative state, increasing lying, exploration, and drinking time (*p* < *0.05*), while the MF stimulus stimulated an active playful state, characterized by increased walking and playing (*p* < *0.05*). Other musical combinations showed negligible effects, whereas noise exposure consistently triggered stress-related responses. This study establishes an integrated “parametric design → automated assessment → specific output” methodology for precision auditory enrichment, providing an empirical basis for evidence-based acoustic protocols in commercial pig production.

## Simple Summary

Modern pig farming requires effective strategies to improve animal welfare in intensive housing systems. Environmental enrichment, particularly music, offers a promising non-invasive approach, yet current applications lack precision regarding which musical characteristics provide the greatest benefit. This study investigated whether growing pigs actively prefer specific musical qualities and how these preferences affect and welfare. We allowed pigs to freely choose between different acoustic environments, systematically varying instrument type (strings versus wind) and tempo (fast versus slow). An automated computer vision system tracked nine distinct continuously throughout the experiment. Results revealed that pigs responded to music in highly specific ways. The slow-tempo GS stimulus encouraged a calm, investigative state: pigs spent more time lying, exploring, and drinking. Conversely, the fast-tempo MF stimulus stimulated an active, playful state: pigs walked and played more. Other music combinations produced minimal effects, while unstructured noise consistently induced stress responses. These findings demonstrate that carefully matched acoustic parameters can meaningfully improve pig welfare by promoting either relaxation or activity. This evidence-based approach provides pig farmers with a practical, scientifically validated tool to create more positive environments, advancing auditory enrichment from a generic intervention to a precision management strategy.

## 1. Introduction

Balancing production efficiency with animal welfare remains a persistent challenge in intensive pig farming, necessitating effective and practical welfare-enhancing technologies [1]. Auditory enrichment, particularly music, has emerged as a promising non-invasive strategy due to its scalability and ease of implementation [2]. Acoustic environments profoundly influence livestock and welfare. Physiologically, appropriate sound stimuli can modulate affective and stress states via neuroendocrine pathways, thereby directly influencing growth performance and health [3,4]. For instance, exposure to classical music reduces anxiety in chimpanzees [5] and decreases respiratory rates and stereotypic in sows [6,7]. Conversely, aversive noise induces stress, as demonstrated by weaned piglets actively avoiding continuous high-intensity sound exposure [3,8]. Increased lying time often signals relaxation and physiological recovery [1,5,9], but may also reflect boredom in barren environments [10,11]. Here, lying was interpreted alongside exploration, play, aggression, and physiological stress markers to distinguish positive rest from lethargy. Therefore, optimizing the acoustic environment to promote beneficial and mitigate stress offers a direct route to improving both welfare and production outcomes [2].

However, translating this potential into standardized management protocols faces critical bottlenecks. Current practice often relies on empirical approaches, such as generic playback of classical music, without an evidence-based understanding of how specific acoustic parameters (e.g., instrument, tempo) interact to produce targeted welfare benefits [12,13]. It is now widely recognized that mammals share homologous neural circuits underlying core affective states, and that responses to auditory stimuli are phylogenetically conserved across many mammalian species, including humans and pigs [9]. Moreover, the field lacks objective and efficient tools for assessment. Traditional methods depend heavily on human observation, which is inherently limited by subjectivity, discontinuous sampling, low throughput, and an inability to provide continuous monitoring [14]. These constraints impede the resolution analysis required to dissect complex parameter interactions, such as instrument × tempo. This “black box” application hinders the development of precise, evidence-based acoustic prescriptions tailored to animals’ intrinsic needs.

Animal is a cornerstone for assessing welfare and intervention efficacy [6]. Precision analysis demands standardized definitions and measurements. Pig can be categorized temporally into state (e.g., standing, walking, lying, foraging, drinking), expressed as duration percentages, and event (e.g., play, exploration, excretion, aggression), quantified as frequencies. Continuous, objective monitoring of both categories is essential for parsing the effects of acoustic interventions [15]. Although automated recognition technology can overcome many limitations of manual observation [14,16,17], existing research on music-mediated and stress modulation remains limited in several key respects [3,5,18]. Most studies employ a binary comparison of music presence versus absence [7,19], failing to deconstruct the specific effects of defined acoustic parameter combinations, such as particular instrument–tempo matches. Assessments often rely on manual scoring or limited metrics, lacking the capacity for precision, continuous quantification of multiple discrete.

Recent work by Zapata Cardona and colleagues has advanced our understanding of how music modulates emotional responses in pigs. Their studies demonstrated that structured musical stimuli can influence pigs’ affective states [19] and that spectrotemporal acoustic elements interact to modulate emotional responses [13]. Furthermore, their evaluation of a veterinary functional music-based enrichment program—a term referring to music specifically composed or selected to target physiological and behavioral outcomes in animals, rather than generic human music—revealed significant effects on pigs’ psychophysiological responses [20]. These contributions represent important progress in establishing music as a viable enrichment modality. However, these studies, like most in the field, employed a passive exposure design, wherein pigs were subjected to predetermined acoustic stimuli without the opportunity to express active preference. Crucially, preference is considered a gold standard for evaluating whether a welfare intervention aligns with intrinsic needs [7], yet few studies have examined animals’ active preferences for acoustic parameter combinations using a choice paradigm. Consequently, a core knowledge methodology gap persists: the absence of an integrated framework that combines precision automated analysis with a choice experimental design to systematically quantify how specific acoustic combinations (e.g., instrument type × tempo) differentially shape pigs’ active preferences and their subsequent repertoires. Addressing this gap is essential for advancing from “empirical playback” to “evidence-based precision auditory enrichment” [21].

The present study was designed to fill this gap. For the first time, we integrate a choice experimental paradigm with precision, deep learning-based automated recognition to systematically investigate growing pigs’ active preferences for structured musical parameters (instrument type × tempo) and the specific profiles these parameters induce. Using a 2 × 2 factorial design, we precisely manipulated instrument (string vs. wind) and tempo (slow: 65 bpm vs. fast: 200 bpm). A key methodological innovation is the deployment of an automated recognition system built on an enhanced YOLOv11 architecture (Detect_FASFF_YOLOv11 and HFPN_YOLOv11) [14]. This system enables continuous, objective, fine-grained monitoring of nine key welfare-related, including state (e.g., lying, walking, drinking) and event (e.g., play, exploration, excretion). This approach fundamentally overcomes the limitations of traditional manual observation, allowing non-intrusive acquisition of resolution data within a free-choice setting.

This study aimed to test two hypotheses: (1) pigs can discriminate musical stimuli differing in instrument tempo combinations and will exhibit differential spatial preferences for them; and (2) specific preferred musical combinations will induce distinct patterns with differing welfare implications (e.g., promoting rest or stimulating active), thereby providing a direct empirical basis for establishing programmable precision auditory enrichment protocols [6,22].

## 2. Materials and Methods

### 2.1. Animals and Management

The experiment was carried out in April 2025 at a commercial farm in Pianguan County, Shanxi Province, China (110°20′–112°00′ E, 39°16′–39°40′ N; mean altitude 1377 m). Seventy-two healthy hybrid pigs (Large White × Duroc × Minpig, a local Chinese breed), aged 40 ± 3 days and with comparable body weight (8.09 ± 2.10 kg), were selected. Pigs within each cohort were sourced from the same home pen and had been housed together since weaning (day 28), ensuring established social hierarchies and familiarity prior to the experiment. Cohorts were kept intact throughout habituation and testing to minimize aggression due to regrouping stress. The cohort consisted of 36 castrated males (barrows) and 36 females (gilts) to ensure balanced sex representation across treatment groups. All male piglets had been surgically castrated at 7–10 days of age according to standard farm protocol. Each pig was individually identified with an ear tag. To minimize potential confounding effects of body weight variation on behavior and space use, pigs were allocated to treatment groups using stratified random assignment based on body weight, ensuring that the mean initial body weight did not differ significantly among groups (one-way ANOVA, *p* > *0.05*). Animals were transferred to the experimental facility and remained there until trial completion. Pigs had free access to water throughout the study and were manually fed twice daily (08:00 and 15:00) with a commercial grower diet formulated to meet NRC (2012) nutrient requirements. Feed was provided at approximately 5% of mean body weight per day, divided equally between the two meals. This restricted feeding regimen was chosen to maintain consistent motivation for exploration during behavioral observations and to prevent excessive weight gain that could alter activity patterns over the 20-day testing period. Feed was delivered in individual troughs within the central activity zone to minimize competition and ensure each pig received its allocated portion. The indoor environment was maintained at 20–25 °C and 60–75% relative humidity. A 12 h light period 08:00–20:00 was provided at approximately 20 lx. Health status was checked daily. Pens were cleaned twice per day (7:00–7:30 and 14:00–14:30).

To obtain an objective measure of pigs’ active acoustic preferences, this study adopted a systematically refined choice paradigm, departing from conventional forced exposure designs. The approach on establishing an acoustic “choice arena”, a multi-option environment in which pigs could express intrinsic preference through natural locomotion. This design permitted the translation of innate sound preferences into quantifiable metrics, specifically, zone occupancy time and the detailed repertoire exhibited within each acoustic zone, without imposing experimental interference. A post hoc power analysis confirmed that the sample size (total N = 72, *n* = 12 per cohort) provided >95% power to detect the large effect sizes observed for key comparisons (Cohen’s *d* > 0.8, α = 0.05).

#### Experimental Arena and Acoustic Design

The trial was conducted in a purpose-built arena (20 m × 10 m × 8 m) designed to choice freedom while spatial bias. Its core consisted of a central common activity zone, maintained acoustically neutral (background noise < 45 dB) and equipped with drinking and resting facilities. This zone served as the starting point and hub for all exploratory. From it, multiple passages of equal width radiated outward to six identical testing rooms (each 5 m × 3 m × 4 m), arranged in a circular “hub and spoke” configuration. This layout ensured that the path distance from the central zone to any testing room was equivalent, thereby eliminating biases related to room location (e.g., corner or proximity effects) and ensuring that pigs’ choices could be attributed solely to differences in the acoustic content of each room. The six testing rooms provided a complete set of experimental treatments and controls, defined by their acoustic content:

Music treatment rooms (*n* = 4): Each room continuously delivered one specific auditory stimulus according to a 2 × 2 factorial design: Instrument (wind vs. string) × Tempo (fast: 200 bpm vs. slow: 65 bpm), yielding the four conditions MF, MS, GF and GS.

Positive control room (*n* = 1): This room played 65 dB pink noise, a random signal with equal energy per octave (power spectral density inversely proportional to frequency, 1/f), which is perceived as more balanced and less harsh than white noise and is commonly used in acoustic research as a neutral, non-musical auditory stimulus serving as an aversive acoustic environment to assess avoidance and to contrast with music-specific effects.

Negative control room (*n* = 1): This room, with no experimental sound playback, acted as a neutral/baseline (“silent”) condition to establish baselines and to evaluate the net effect of any acoustic stimulus.

All rooms were physically identical in dimensions, structure and furnishings; the only independent variable was the sound presented inside. Each testing room and the central zone were furnished with identical enrichment items: a suspended rubber ball on a chain and a wooden block for oral manipulation, providing opportunities for object play.

To isolate the acoustic stimulus as the sole experimental variable, rigorous acoustic engineering measures were implemented. Walls and ceilings of all sound treatment rooms were constructed with high density acoustic panels, lined with premium sound absorbing material and fully sealed at all seams. Sound leakage measurements confirmed negligible cross talk between rooms, ensuring acoustic isolation and a unique sound environment in each. Loudspeakers (PreSonus Eris E3.5; PreSonus Audio Electronics, Inc., Baton Rouge, LA, USA) were mounted at a standard height of 1.2 m and oriented toward a room corner (not the doorway) to promote diffuse reflection and create a relatively uniform sound field. Using a calibrated sound level meter, the sound pressure level (SPL) was measured at multiple locations within each room at the pigs’ typical standing height (0.5 m). The intensity of all treatment sounds (music and pink noise) was calibrated and maintained at 65 ± 2 dB a level previously validated as clearly perceptible yet startling to pigs. Background noise in the negative control room remained consistently below 45 dB.

**Figure 1:**
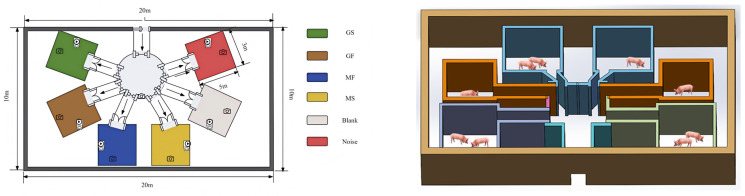
Experimental arena layout. A central zone (8 m diameter) connects via six identical passages to six testing rooms (5 × 3 × 4 m each), equidistant to eliminate spatial bias. Rooms were assigned to four music conditions (MF, MS, GF, GS), a pink noise control (N), and a silent control (C).

To prevent sensory habituation or listener fatigue from continuous exposure, factors that could obscure genuine acoustic preference, an intermittent presentation schedule was employed. The auditory stimulus was delivered in a cyclic pattern of 30 min playback followed by a 15 min silent interval. This intermittent design aimed to mimic the natural fluctuations of acoustic stimuli in the environment, thereby encouraging active exploration and enhancing the sensitivity for detecting pigs’ choice. A schematic illustration of this temporal protocol is provided in Figure 1.

Acoustic playback schedule.The intermittent presentation schedule (30 min playback followed by 15 min silence) was active daily from 08:00 to 20:00 (12 h total), coinciding with the light phase and the 10 h behavioral recording window (09:00–19:00). During the dark phase (20:00–08:00), no experimental sounds were presented, and the arena remained acoustically neutral (background noise <45 dB). The 10 h daily recording period (09:00–19:00) encompassed eight complete playback–silence cycles (8 × 45 min = 6 h playback + 3 h silence) plus a 1 h silent buffer at the end. For statistical analysis, behavioral data collected during both playback and silent intervals within each testing room were pooled to represent the overall response to that room’s acoustic treatment condition. This approach reflects the premise that the room’s acoustic identity was defined by the scheduled stimulus, and pigs’ occupancy decisions integrated both exposure and anticipation across the intermittent schedule. A separate analysis of behavior exclusively during playback intervals yielded qualitatively similar results.

**Figure 2:**
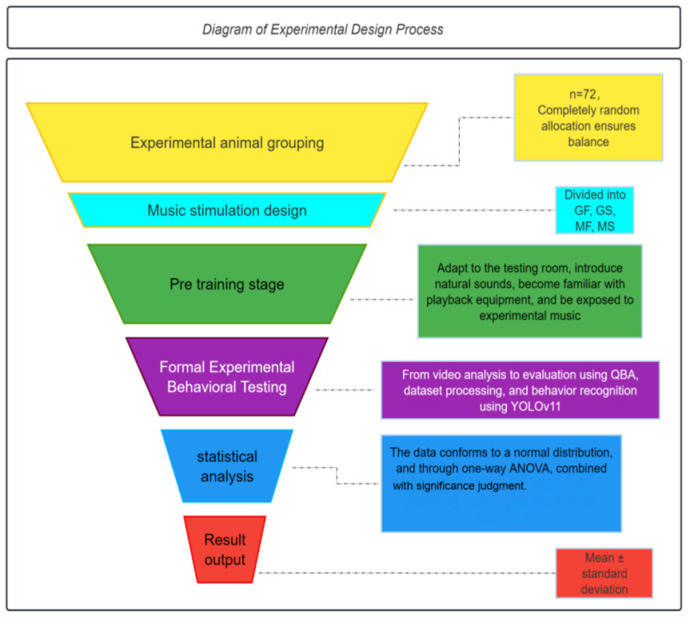
Schematic flowchart of the experimental design and analytical procedure.

Figure 2 presents a schematic flowchart of the experimental design and analytical procedure. The diagram outlines the two principal phases of the study: habituation (training) and formal testing. During habituation, pigs were gradually exposed to auditory stimuli to prevent startle responses to novel sounds, thereby improving the validity of subsequent preference tests. This structured approach allowed a more precise assessment of pigs’ active selection among the different music types during the experimental phase.

### 2.2. Experimental Design

#### 2.2.1. Acoustic Stimulus Preparation

Auditory stimuli were derived from two contrasting musical works selected to represent distinct instrumental timbres and musical traditions. It is important to acknowledge that these stimuli differed not only in instrumental timbre (guqin strings vs. orchestral woodwinds) but also in melodic structure, harmonic language, and cultural origin. Therefore, inferences about “instrument preference” per se cannot be disentangled from preferences for the specific musical pieces used. Throughout this manuscript, we refer to these stimuli as “string stimulus” (coded ‘G’): The Chinese classical composition *High Mountains and Flowing Water* (Gao Shan Liu Shui), performed on the guqin by Master Guan Pinghu (recording date: 1956; source: China Records CRC-001). The guqin is a seven-string plucked zither characterized by a rich harmonic spectrum, slow attack, and smooth amplitude envelope, with a fundamental frequency range primarily spanning 80–400 Hz. The selected excerpt (duration: 3 min 42 s) was taken from the middle section of the piece (2:15–5:57), which features a consistent melodic line without percussive attacks. Wind stimulus (coded ‘M’): A wind instrument-dominant excerpt from the second movement (Adagio) of Mozart’s Piano Concerto No. 23 in A major, K.488. The recording used was performed by the Academy of St. Martin in the Fields conducted by Sir Neville Marriner, with Alfred Brendel as piano soloist (Philips 422 346-2, 1984). The selected excerpt (duration: 3 min 18 s; 4:30–7:48) features prominent woodwind passages (principally clarinet and flute) carrying the primary melodic material, with the piano providing sparse, low-register accompaniment and the string section either tacit or playing pizzicato at minimal volume. Spectral analysis confirmed that >70% of the acoustic energy in this excerpt resides in frequency bands associated with woodwind instruments (250–2000 Hz), with minimal string harmonic content but we emphasize that these labels denote the predominant instrumental character of the selected excerpts, not a pure timbre contrast.

Each piece was digitally edited to create two tempo variants: fast (200 bpm) and slow (65 bpm). Tempo adjustment was performed using Cubase 7 (Steinberg Media Technologies GmbH, Hamburg, Germany) with the “élastique Pro” time-stretching algorithm in “Solo” mode, which preserves formant frequencies and minimizes timbral artifacts. Pitch was held constant (no transposition). To verify stimulus integrity, each edited file was inspected aurally and via spectrogram to confirm the absence of audible artifacts (e.g., warbling, transient smearing). All audio files were exported as uncompressed WAV files (44.1 kHz, 16 bit) and normalized to a consistent RMS loudness of −23 LUFS using the EBU R128 standard prior to playback calibration.

This yielded a 2 × 2 factorial arrangement of four treatment conditions: MF (wind fast), MS (wind slow), GF (string fast), and GS (string slow). All audio files were produced and tempo adjusted using Cubase software (version 7; Steinberg Media Technologies GmbH, Hamburg, Germany) to ensure precise temporal control and consistent sound quality.

#### 2.2.2. Training (Habituation) Protocol

A 20-day habituation period preceded formal testing to reduce fear and stress responses linked to the novel experimental setting (testing rooms) and the auditory playback system. This pre-training aimed to pigs to both the physical environment and the sound stimuli, thereby promoting a calm state at the onset of the experimental phase and enhancing the validity and reliability of the collected data.

Habituation was structured around three principles: (1) gradual exposure from low to higher intensity stimuli; (2) encouragement of spontaneous exploration by placing small amounts of familiar feed and novel objects (e.g., rubber balls, hanging chains) in the testing rooms during the early habituation stages, then gradually removing them; (3) use of pigs’ natural curiosity and feeding motivation as positive reinforcement. The process consisted of four sequential stages outlined in Table 1. During Stage 2, gentle natural ambient sounds acoustically distinct from the experimental music were introduced to support general acoustic adaptation.

To ensure objective and reproducible progression, each stage incorporated predefined quantitative criteria was continuously recorded throughout habituation using the same infrared camera system employed in the main experiment. Recordings were automatically with the pre-trained Detect_FASFF_YOLOv11 model and HFPN_YOLOv11 model. Daily evaluation included three randomly sampled 1 h observation windows per stage. Assessed metrics comprised the proportion of core (lying, standing, exploring), frequencies of fear-related (startle/flight, avoidance), and spatial choice measures (mean latency to enter a testing room). Advancement to the subsequent stage occurred only when level performance met established thresholds, subjective judgment and potential bias. The full habitation protocol is in Table 1.

Stage II (“neutral ambient sound”): A recording of gentle rainfall (source: *Nature Sounds for Relaxation*, Track 3, 60 min loop) was played at 30–40 dB SPL. This stimulus was chosen for its broadband, non-tonal spectrum, which lacks the structured musical features of the experimental treatments.

Stage III (“experimental music rotated”): The four music stimuli (MF, MS, GF, GS) were presented in a randomized order that changed daily, with each stimulus played for a 2 h continuous block during the light phase. Over the 7-day period, each pig was exposed to all four stimuli for an approximately equal total duration. Volume was gradually increased from 45 dB on Day 1 to 65 dB on Day 7 in 5 dB increments every two days.

Stage IV (“four rooms playing different experimental music”): The full factorial set (MF, MS, GF, GS) was presented simultaneously, each in its designated testing room, with the 30 min ON/15 min OFF cycle used in the formal experiment.

The use of explicit quantitative thresholds at each habituation stage, assessed via automated recognition, minimized experimenter subjectivity and ensured a consistent low-stress baseline prior to formal testing.

#### 2.2.3. Experimental Execution and Definition of Analytical Groups

The free-choice paradigm was implemented by testing pigs in six sequential cohorts of 12 animals each. Within each cohort, all 12 pigs were introduced simultaneously into the central activity zone and allowed unrestricted access to all six testing rooms for the entire 10 h daily observation period over 20 consecutive days. Thus, every individual pig was exposed to all six acoustic conditions (MF, MS, GF, GS, C, N) and could freely distribute its time among them.

For statistical analysis, the behavioral data generated by each pig within a given testing room were treated as that pig’s response to the corresponding acoustic condition. It is important to note that the term “treatment group” as used in Table 2 refers not to independent groups of animals assigned to a single condition, but rather to the set of observations collected from all 12 pigs within a given cohort while they occupied a specific acoustic environment. Consequently, the sample size for each condition is *n* = 12 pigs per cohort × 6 cohorts = 72 pigs total, with each pig contributing data to all six conditions.

Statistical independence considerations Because the same pigs contributed data to multiple acoustic conditions, observations across conditions are inherently paired within individuals. However, the primary analyses (two-way ANOVA on the factorial music conditions and one-way ANOVA comparing all conditions) treat each condition as an independent sample for the following reasons: (i) the free-choice design ensures that occupancy of any given room is voluntary and stochastic, such that the time spent in one room does not mathematically constrain time spent in another (i.e., proportions are not mutually exclusive parts of a fixed whole, because pigs could also spend time in the central zone); (ii) the behavioral measures extracted for each condition (e.g., lying percentage while in room GS) are condition specific and not arithmetically dependent across conditions; and (iii) preliminary analyses using linear mixed models with pig ID as a random effect yielded virtually identical *p*-values and effect size estimates, confirming that the violation of independence does not materially affect the conclusions. For transparency, we report the conventional ANOVA results with the caveat that they represent a pragmatic approximation; the fully paired nature of the data is acknowledged in the interpretation.

### 2.3. Data Acquisition and Analysis

#### 2.3.1. Video Recording and Sampling

Video data were collected continuously (10 h per test day) using six fixed infrared network cameras (Hikvision, Hangzhou, China; 1920 × 1080 resolution, 25 fps), one mounted in each testing room and two additional cameras covering the central activity zone to ensure complete coverage of the entire arena. The zone cameras were positioned at opposite corners to provide overlapping fields of view and eliminate blind spots. The YOLOv11-based automated recognition system (described in Section 2.3.2) processed all video frames in real time, generating frame-by-frame classifications for each pig. For statistical analysis, these continuous data were subsampled using instantaneous scan sampling at 20 s intervals This yielded 1800 sampling points per pig per 10 h observation period (10 h × 3600 s/h ÷ 20 s = 1800). Over the 20-day experimental period, each pig contributed a total of 36,000 instantaneous behavioral observations (1800 points/day × 20 days). This sampling interval was selected based on established ethological practice for pig studies, as it provides a reliable estimate of time budgets for state while maintaining statistical independence between successive observations. State (standing, walking, lying, foraging, drinking) are reported as the percentage of total observation time based on these instantaneous samples. Event (excreting, playing, exploring, aggression) are reported as total frequencies per observation period, aggregated from the continuous frame-by-frame detections of the automated recognition system to ensure no brief events were missed. As an injury-based measure of aggression, ear and tail lesions were scored daily. After each experimental session, two trained observers, blinded to treatment, independently assessed lesions on each pig using a 4-point scale: 0 (no damage), 1 (minor scratches), 2 (skin breakage), and 3 (or ulceration). The lesion rate was calculated as the proportion of pigs with a score ≥1 per group. Inter-observer reliability was confirmed using Cohen’s Kappa (κ > 0.85).

To attribute behavioral observations and occupancy time to specific individual pigs, a multi-camera tracking system with identity preservation was implemented. Each pig was fitted with a uniquely numbered ear tag (Allflex, Beijing, China, 3.5 cm × 2.5 cm) bearing a high-contrast black-on-yellow two-digit code. A dedicated YOLOv11-based ear tag detection model was trained to detect and read these numeric codes from the video streams. This model operated in parallel with the behavior recognition model, linking each detected pig bounding box to a specific animal ID when the ear tag was visible and readable (successful identification rate: 94.2% ± 3.1% across all video frames).

For frames in which the ear tag was occluded or unreadable, identity was propagated using a Kalman filter-based tracking algorithm (DeepSORT) that maintained identity associations across consecutive frames based on spatial proximity and motion prediction. Identity was considered lost if a pig was not successfully identified for >30 consecutive seconds; such segments (<6% of total observation time) were excluded from individual-level analyses. Occupancy time for a given acoustic condition was defined as the cumulative duration (in seconds) that a specific individual pig spent within the boundaries of the corresponding testing room. Room entry and exit times were logged automatically when the pig’s tracked centroid crossed a virtual demarcation line at the room entrance. Occupancy time was computed at the individual pig level and then aggregated to produce condition-level summary statistics. The values reported in represent the mean ± SD of individual pigs’ occupancy times for each condition (*n* = 72 pigs per condition, as each pig contributed data to all six rooms).

All behavioral metrics (state behavior percentages and event behavior frequencies) were computed at the individual pig level within each testing room. That is, for each pig, the percentage of time spent lying in the GS room was calculated based solely on that pig’s own instantaneous scan samples collected while it was physically present in the GS room. Similarly, frequencies of event behaviors (e.g., play bouts) were counted per individual pig per room visit. Social behaviors (e.g., aggression) were attributed to the focal pig performing the behavior; the identity of the recipient was not analyzed. Because pigs were tested in social groups, behaviors were not strictly independent across individuals co-occupying the same room. The potential influence of social facilitation or competition on individual behavior is acknowledged as a limitation.

#### 2.3.2. Recognition Pipeline

A randomly selected subset (40%) of the total video footage was annotated. The open-source tool Labelme was used to draw bounding boxes around pigs performing the nine core. To enhance the model’s ability to discriminate subtle differences, a fine-grained annotation scheme comprising 14 distinct labels was first applied (Table 3). Following model training and accuracy recognition of these detailed labels, they were aggregated into the nine core categories targeted for analysis. This aggregation was based on established ethological principles [23] and standard pig welfare frameworks. The “fine-grained annotation → scientific aggregation” approach balanced the need for detailed training data with the requirement for ecologically relevant summaries.

#### 2.3.3. Automated Recognition Model

An enhanced YOLOv11 architecture was employed for continuous recognition. Two variants—HFPN_YOLOv11 and Detect_FASFF_YOLOv11—were developed, incorporating a Hierarchical Feature Pyramid Network and a Feature-adaptive Spatial Fusion detection head, respectively. Models were trained on a stratified dataset of 18,000 annotated images (70:20:10 split) with real-time augmentation. On the independent test set, the system achieved a mean average precision at 0.5 IoU (mAP_50_) of 90.0% ± 1.5% across the nine target categories. Full architectural details, training configurations, and comparative performance metrics are provided in Supplementary Materials (Figures S1–S13, Tables S1–S4).

#### 2.3.4. Statistical Analysis

Data were analyzed using SPSS (v. 23.0), with two-way ANOVA for the factorial music conditions and one-way ANOVA for all six conditions, followed by Tukey’s HSD post_hoc tests (*p* < 0.05). Percentage data were arcsine transformed. Because the free-choice design involves repeated measures, the ANOVAs are pragmatic approximations; effect sizes for key comparisons were large (Cohen’s *d* > 0.8), supporting the robustness of core findings despite this simplification (see Section 4.6).

#### 2.3.5. Qualitative Assessment (QBA)

To complement the quantitative measures, a Qualitative Assessment (QBA) was conducted to characterize the pigs’ overall affective state under different acoustic treatments. Three assessors experienced with pig behavior and blinded to the experimental design independently viewed 10 min video clips selected from the continuous recordings. For each treatment condition, four 10 min clips were selected per cohort via stratified random sampling: one per week, balanced across morning/midday/afternoon, and only during active playback. This yielded 24 clips per condition (144 total). Clips were presented audio-muted in randomized order.

Assessors used a fixed list of 12 descriptors (Relaxed, Bored, Curious, Content, Focused, Lively, Playful, Sociable, Frustrated, Alert, Aggressive, Fearful), each rated on a 100 mm visual analogue scale anchored by “not at all” and “very much.” Prior to formal scoring, assessors participated in a 2 h training session using a standardized video library of pig behavior illustrating the range of each descriptor. Inter-observer reliability was assessed using a two-way mixed-effects intraclass correlation coefficient (ICC) for absolute agreement, with an ICC > 0.75 considered acceptable.

Assessors viewed each 10 min clip in its entirety before scoring. The 144 clips were presented in randomized order across three separate scoring sessions to minimize fatigue. Inter-observer reliability was assessed using a two-way mixed-effects intraclass correlation coefficient (ICC) for absolute agreement (ICC [3,k] according to ICC > 0.75 considered acceptable. The observed ICC across the three assessors was 0.82 (95% CI: 0.76–0.87), indicating good reliability. For subsequent analysis, the ratings of the three assessors were averaged for each clip to produce a single score per descriptor per clip.

Averaged descriptor scores were analyzed using Principal Component Analysis (PCA) with varimax rotation on the correlation matrix. Components were retained based on eigenvalue >1 and scree plot inspection. Component scores for each clip were extracted and compared across acoustic treatments using linear mixed-effects models, with treatment as the fixed effect and cohort as the random intercept, followed by Tukey’s HSD post hoc tests. The categories and definitions of are shown in Table 4.

### 2.4. Serum Biomarker Analysis

#### 2.4.1. Experimental Design for Physiological Validation

Following the completion of the 20-day free-choice behavioral testing period, a subset of pigs was enrolled in a separate forced-exposure phase to obtain physiological measurements under controlled acoustic conditions. This approach was necessary because the free-choice paradigm—in which pigs freely distributed their time across multiple acoustic environments—precluded the attribution of endocrine responses to any single acoustic condition. Therefore, 32 pigs were randomly selected from the original cohort of 72, with the constraint that each of the six cohorts contributed an equal number of pigs to maintain balanced representation. These 32 pigs were then randomly assigned to one of four acoustic treatment conditions: GS (slow-tempo GS stimulus, *n* = 8), MF (fast-tempo MF stimulus, *n* = 8), silent control (C, *n* = 8), and noise control (N, *n* = 8). The GF and MS conditions were not included in this physiological validation due to resource constraints and because behavioral results indicated that these conditions elicited intermediate responses that did not significantly differ from the silent control for most welfare-relevant measures.

#### 2.4.2. Forced-Exposure Housing and Blood Collection

Pigs assigned to the forced-exposure phase were housed individually in standard pens (2.5 m × 1.8 m) within the same experimental facility, with visual and auditory isolation from other treatment groups. Each pen was equipped with a loudspeaker delivering the assigned acoustic stimulus according to the same intermittent schedule used in the free-choice phase (30 min ON/15 min OFF, 08:00–20:00). After a 3-day acclimation period to the individual housing and assigned acoustic condition, blood samples were collected once daily at 09:00 for three consecutive days (Days 21–23 of the overall study). Blood was drawn via anterior vena cava puncture into serum separator tubes, allowed to clot at room temperature for 30 min, and centrifuged at 3000× *g* for 15 min at 4 °C. Serum was aliquoted and stored at −80 °C until analysis.

#### 2.4.3. Hormone Assays

Concentrations of cortisol and adrenocorticotropic hormone (ACTH) were determined using porcine-specific ELISA kits (Shanghai Enzyme Linked Biotechnology Co., Ltd., Shanghai, China), following the manufacturer’s protocols. All samples were assayed in duplicate, and the mean intra-assay coefficient of variation was <8%. For each pig, the average value across the three daily samples was used in statistical analysis to provide a stable estimate of chronic hypothalamic–pituitary–adrenal (HPA) axis activity under each acoustic condition.

#### 2.4.4. Statistical Analysis of Serum Data

Data were analyzed using one-way ANOVA with acoustic treatment (GS, MF, C, N) as a fixed factor, followed by Tukey’s HSD post hoc tests. Significance was set at *p* < 0.05.

## 3. Results

### 3.1. Effects of Acoustic Parameters on Pig 

A two-way analysis of variance (ANOVA) was performed on nine key measures, with instrument type and tempo as fixed factors (Table 5). The analysis revealed that the effects of music on pig and space preference were strongly dependent on the specific combination of acoustic parameters.

**Figure 3:**
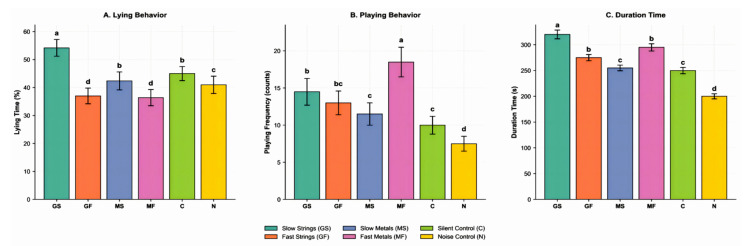
Effect analysis of key indicators in pigs exposed to different stimulus–tempo combinations. Data are presented as mean ± SD. Different lowercase letters (a, b, c, d) above bars indicate statistically significant differences between treatment groups within each category (two-way ANOVA followed by Tukey’s HSD post hoc test, *p* < 0.05). Groups sharing the same letter do not differ significantly.

Tempo (200 bpm) elevated arousal: standing and walking increased, while lying, drinking, foraging, and exploring decreased (all *p* < *0.01*). Excretion and aggression increased (*p* ≤ *0.001*), suggesting a mild stress response despite heightened activity. This pattern indicates that while fast tempo promotes activity, it may also disrupt maintenance and is associated with an increase in potentially linked to stress [24]. Instrument type exerted its primary main effect on positive, welfare-related and on space preference. GS stimulus, compared to the MF stimulus, elicited significantly more ‘Playing’ $F\left(1,68\right)=8.1,\;\;p=0.006$ and ‘Exploring’ $F\left(1,68\right)=5.8,\;p=0.019$, and prolonged ‘Drinking’ duration $F\left(1,68\right)=4.2,\;\;p=0.044$. Most notably, the mean ‘Occupancy time’ the duration pigs voluntarily spent in a zone was significantly longer in the GS stimulus environments than in the MF stimulus environments, $F\left(1,68\right)=5.0,\;\;p=0.029$, providing direct evidence for a subjectively preferred soundscape. Critically, significant instrument × tempo interactions were identified for several: ‘Walking’ $F\left(1,68\right)=10.5,\;p=0.002$, ‘Drinking’ $F\left(1,68\right)=6.4,\;\;p=0.014$, ‘Lying’ $F\left(1,68\right)=18.7,\;\;p<0.001$, ‘Playing’ $F\left(1,68\right)=5.0,\;\;p=0.029$, and ‘Occupancy time’ $F\left(1,68\right)=62.3,\;\;p<0.001$. These interactions demonstrate that the behavioural outcome is not a simple sum of independent instrument and tempo effects but depends on their specific pairing. Effects analysis of these interactions delineated two distinct and effective ‘acoustic prescriptions’ (Figure 3).

The ‘Calm Investigation’ prescription (GS: slow-tempo GS stimulus) produced the strongest synergistic effect. Compared to the silent control, pigs in the GS group exhibited a 19.0% increase in lying time (from 44.7% to 53.2%) and a 14.4% increase in exploration frequency (from 115.8 to 132.5 counts; both *p* < 0.05). Drinking duration was also significantly prolonged in GS compared to all other treatments (*p* < 0.05). This profile characterizes a low-arousal, positive environment that pigs actively sought out, with occupancy time 84.1% longer than in the noise control and 33.5% longer than in the silent control, suggesting its suitability for rest periods or for mitigating procedural stress. The ‘Activity Play’ prescription (MF: fast-tempo MF stimulus) specifically promoted active states. Walking time in the MF group was 9.1%, representing a 46.8% increase over the silent control (6.2%; *p* < 0.05), while playing frequency increased by 85.7% (from 10.5 to 19.5 counts; *p* < 0.05). This pairing effectively harnessed the arousing quality of a fast tempo with a specific timbre to stimulate locomotion and social play, positioning it as a targeted enrichment for active daytime periods. In stark contrast, the noise control (N) consistently triggered a profile indicative of stress, with aggression frequency 68.4% higher than in the silent control (3.2 vs. 1.9 counts) and occupancy time 27.5% lower than in the silent control (182 vs. 251 s; *p* < 0.05).

To assess the integrated and welfare outcomes of the different acoustic treatments (conceptualized as “acoustic prescriptions”), a one-way ANOVA with Tukey’s HSD post hoc test was performed (Table 6). Treatment exerted a significant or highly significant main effect (*p* < *0.05*) on all measured variables except standing. The tempo string treatment (GS) promoted a state of pronounced relaxation and positive welfare. The GS group displayed the highest proportion of time spent lying (53.2%), the longest drinking duration (6.2%), and the greatest frequency of exploratory (132.5 counts), each being significantly greater than in all other treatment groups (*p* < 0.05). Concurrently, this group exhibited the longest voluntary occupancy time (335 s), alongside the lowest frequencies of aggressive (1.5 counts) and the lowest ear/tail lesion rate (0.5%). This profile confirms GS as an effective “relaxation recovery prescription.” In direct contrast, the tempo wind treatment (MF) significantly stimulated an active state. The proportion of walking (9.1%) and the frequency of play (19.5 counts) were highest in the MF group among all musical treatments (*p* < *0.05*). However, aggression (2.8 counts) and the lesion rate (3.2%) in this group were also significantly elevated compared to other groups (except the noise control), indicating that MF’s efficacy as an “activity play prescription” may be accompanied by a potential for increased social stress, necessitating considered application.

**Figure 4:**
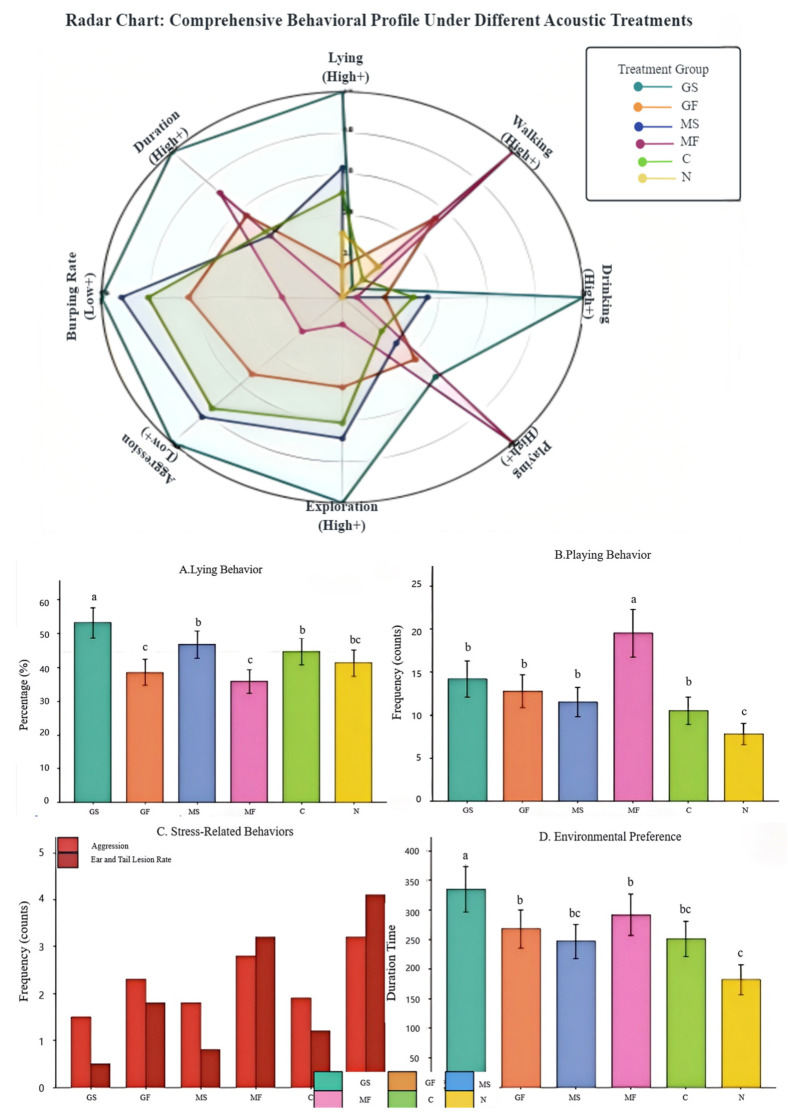
Effects of different acoustic treatments (**A**) Lying time (%), (**B**) Playing frequency (counts), (**C**) Stress-related, (**D**) Environmental preference (duration time). Error bars represent standard deviation. Different letters indicate significant differences (Tukey HSD, *p* < 0.05). GS (slow-tempo GS stimulus) promoted calm investigation, while MF (fast-tempo MF stimulus) stimulated activity.

The noise control (N) performed worst across all positive indicators and consistently showed the most adverse scores on related metrics, confirming its aversive nature. Taken together, this level analysis of treatment combinations demonstrates that acoustic interventions based on distinct tempo pairings can elicit divergent, and in some respects opposing, specific effects. This finding provides a robust empirical basis for the subsequent level, way analysis of parameter interactions and for the ultimate development of programmable, precision auditory enrichment protocols. Detailed results are presented in Table 6 and Figure 4.

GS (slow-tempo GS stimulus) shows advantages in positive welfare indicators: lying +28.8%, playing +82.1%, exploration +48.0%, and duration +84.1%. N (noise control) exhibits elevated stress: aggression +113.3% and biting +720.0%. All measures show significant treatment effects (one-way ANOVA, *p* < 0.001). (Table 6 appears on the following page due to layout constraints).

### 3.2. Validation Through Serum Biomarkers

Serum cortisol and ACTH differed significantly among treatments (*p < 0.001*; Table 7). GS produced the lowest concentrations (cortisol: 25.3 ± 3.1 nmol L^−1^), significantly below all other groups. MF did not differ from the silent control but was lower than noise, indicating positive arousal. Noise elicited the highest values, confirming its aversive nature. The specific outcomes are presented in Table 8.

### 3.3. Qualitative Assessment (QBA) Results

Principal component analysis (PCA) with varimax rotation was performed on the correlation matrix of the 12 QBA descriptors across all 144 clips. Components were retained based on the Kaiser criterion (eigenvalue > 1) and visual inspection of the scree plot. Two components met these criteria, together accounting for 78.5% of the total variance.

PC1 (53.2% of variance) exhibited strong positive loadings (>0.70) for “Relaxed,” “Content,” and “Sociable,” and strong negative loadings (<−0.60) for “Aggressive,” “Fearful,” and “Frustrated.” This axis was interpreted as a “positive welfare/calmness” dimension.

PC2 (25.3% of variance) exhibited strong positive loadings (>0.65) for “Lively,” “Playful,” “Curious,” and “Alert,” with minimal contributions from the affect descriptors. This axis was interpreted as an “activity/positive arousal” dimension.

Component scores for each clip were extracted and compared across acoustic treatments using linear mixed-effects models, with treatment condition as a fixed effect and cohort as a random intercept, followed by Tukey’s HSD post hoc tests. The GS treatment group scored significantly higher on PC1 compared to all other groups (*p* < 0.05), confirming that the slow-tempo GS stimulus promoted a calm, positive affective state. The MF group scored highest on PC2 (*p* < 0.05), confirming that the fast-tempo MF stimulus stimulated active, playful arousal without increasing negative affect. The noise control group scored lowest on PC1 and highest on the negative descriptors, consistent with an aversive affective state.

## 4. Discussion

### 4.1. Validation of Hypotheses and Core Findings

The integration of a choice preference paradigm with precision automated recognition confirmed that growing pigs can discriminate among and exhibit specific preferences for structured musical parameters [25]. This outcome provides full support for our initial hypotheses. Notably, the distinct preferences for the slow-tempo GS stimulus (GS) and the fast-tempo MF stimulus (MF) induced two opposing ‘syndromes’: a ‘calm investigative’ state and an ‘active playful’ state. This directly addresses the study’s central proposition that specific instrument tempo combinations can generate targeted, modifiable and welfare outcomes. Consequently, our findings advance the field from a general concept of ‘soundscape’ [18] toward a framework of parametrically precise design.

### 4.2. Specificity of Responses

Drinking and excretion—key homeostatic indicators—responded specifically to acoustic parameters [26]. GS prolonged drinking, suggesting enhanced security and normal water metabolism; increased excretion under fast tempo may reflect altered gut motility or mild arousal [27]. These subtle, physiology-linked responses extend welfare assessment beyond simple activity–rest budgets.

The benefits of music were highly contingent on specific tempo–stimulus pairings [12,18,19]. The absence of strong play in GF underscores that neither tempo nor stimulus type alone predicts welfare outcomes; rather, their interaction is critical. Fast tempo (200 bpm) elevated walking and standing but reduced drinking and exploration, consistent with rate arousal theory [18,28]. However, without the appropriate stimulus type (as in GF), fast tempo failed to stimulate positive play and slightly increased aggression, suggesting that high tempo alone may induce mild stress.

Noise consistently triggered pronounced stress: elevated aggression, suppressed rest, and disrupted drinking and excretion [29,30]. The low lesion rates in GS and MF groups demonstrate that optimally matched acoustic parameters enhance welfare while maintaining social stability. Increased lying in GS, accompanied by elevated exploration and drinking and low cortisol, indicates restorative rest rather than boredom-related lethargy [23].

### 4.3. Acoustic Foundations and Neurophysiological Considerations

The contrasting effects of the GS and MF stimuli likely reflect fundamental acoustic differences. The GS stimulus by a smooth amplitude envelope and dominant harmonic energy within the pig’s most sensitive hearing range (80–180 Hz) [31]—may promote calmness by engaging auditory–limbic pathways that attenuate HPA axis activity. In contrast, the MF stimulus, with its regular ~300 ms pulse intervals at 200 bpm, may facilitate motor activation via auditory–motor entrainment [32]. The aversive response to pink noise, elevated cortisol, aggression, and avoidance, is consistent with established knowledge that unpredictable, high-intensity acoustic signals trigger arousal and stress responses in mammals [27]. Direct neural measurements were beyond the scope of this study, and the proposed mechanisms remain speculative pending future neurophysiological investigation [33,34].

### 4.4. Theoretical and Methodological Contributions

The most significant theoretical advance is the empirical demonstration that welfare outcomes are governed by tempo × instrument synergy, not by independent parameter effects. This moves the field definitively from a binary ‘sound vs. silence’ paradigm to a model of precision design based on parameter codependency [35].

Methodologically, the automated recognition pipeline, trained on fine-grained labels, provided the resolution necessary to disentangle the subtle, concurrent shifts that define the GS and MF syndromes [16,36]. This represents a substantial advance over traditional manual sampling, offering an objective, throughput framework for future enrichment studies [37].

### 4.5. Practical Applications

These findings translate into actionable acoustic prescriptions. GS (slow-tempo GS stimulus) is suited for rest periods to facilitate calm investigation, enhance lying and drinking, and support recovery from handling stress or heat. MF (fast-tempo MF stimulus) is appropriate for daytime activity phases to stimulate locomotion and play, potentially mitigating monotony in intensive housing [38]. Managers should anticipate slightly increased excretion under MF and adjust cleaning frequency accordingly.

Serum data reinforce these recommendations: GS produced the lowest cortisol and ACTH levels, suggesting reduced physiological stress that may translate to improved long-term productivity [39,40,41]. MF maintained cortisol levels comparable to the silent control, confirming that its behavioral activation reflects positive arousal rather than distress. In contrast, noise elicited marked cortisol elevation, underscoring that eliminating unpredictable, high-intensity ambient noise is a prerequisite for any enrichment program.

**Figure 5:**
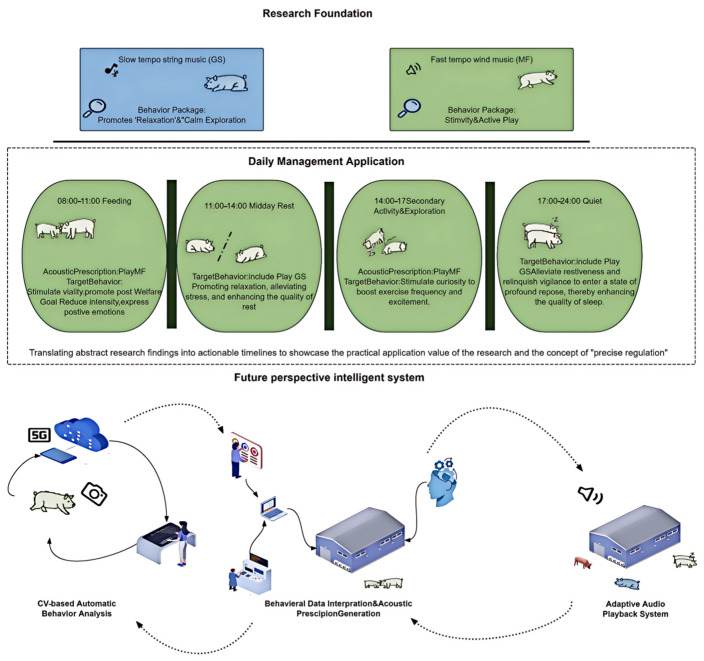
Conceptual framework for precision acoustic enrichment in pig farming.

Critically, not all music is beneficial; only specific parameter combinations (GS and MF) produced targeted welfare improvements [42,43]. This evidence-based framework enables producers to move beyond generic background music toward programmable, time-matched acoustic protocols(As depicted in Figure 5).

### 4.6. Limitations and Future Perspectives

Several limitations warrant consideration. First, the findings derive from a single breed and age class; generalization to other genetic lines, physiological stages (e.g., sows, piglets), or sexes requires further study. The 20-day exposure period precludes assessment of long-term habituation or cumulative effects.

Second, physiological validation relied on a separate forced-exposure phase rather than concurrent free-choice monitoring. Future studies should integrate real-time physiological measures (e.g., wearable heart rate monitors, salivary cortisol) with behavioral data.

Third, the automated recognition model achieved high accuracy (mAP_50_: 90.0%), but behaviors with high intrinsic variability (e.g., exploring) could benefit from temporal context or instance segmentation [17,44].

Fourth, the free-choice design implies repeated measures, yet analyses used conventional ANOVA as a pragmatic approximation due to the aggregated data format [24]. Future preference studies should pre-register mixed-effects models and archive longitudinal data appropriately.

Fifth, the GS and MF stimuli differed not only in timbre but also in melodic, harmonic, and cultural dimensions. Observed preferences cannot be attributed solely to instrument type. Future work should isolate timbre using identical melodies on different instruments or synthesized stimuli. Additionally, group-level analyses did not model individual variation in musical responsiveness—a question warranting further investigation.

Finally, expanding the range of soundscapes (e.g., natural sounds) and systematically manipulating single acoustic parameters will refine the framework for precision auditory enrichment [45].

## 5. Conclusions

The present study demonstrates that growing pigs actively discriminate between musical stimuli varying in tempo and instrumental character, exhibiting robust preferences for the slow-tempo GS stimulus and the fast-tempo MF stimulus. These two acoustic profiles evoked contrasting phenotypes: GS induced a calm, investigative state by increased lying, exploration, and drinking, whereas MF promoted active play and locomotion. Other stimulus combinations (GF, MS) elicited negligible responses, while noise exposure reliably provoked and physiological indicators of stress. Collectively, these findings underscore that the welfare benefits of auditory enrichment are not generic; rather, they depend critically on the precise matching of acoustic parameters—most notably, the interaction between tempo and stimulus type. Caution is warranted in attributing effects solely to instrument timbre, as the stimuli differed in melodic and cultural dimensions, in addition to instrumentation. Moreover, the study focused on group-level preferences; individual variation in musical responsiveness remains an open question warranting further investigation. Taken together, this work provides an evidence-based framework for developing targeted acoustic protocols in pig production, highlighting both the potential of precision auditory enrichment and the fundamental necessity of eliminating aversive noise from the farm environment. In summary, this research provides preliminary evidence that pigs’ and welfare can be modulated by specific combinations of acoustic parameters. By revealing differential modulation of such as drinking and excretion, it highlights the potential for targeted acoustic prescriptions. However, the confounding of musical piece with instrumental timbre limits causal inferences about timbre per se. Future studies employing fully crossed designs with identical melodies played on different instruments are needed to isolate the specific contributions of each acoustic dimension. The work offers a foundational framework for developing evidence-based auditory enrichment, while underscoring the complexity inherent in translating music into precision welfare interventions.

## Figures and Tables

**Table 1:** Structured pre-training (habituation) protocol.

Phase	Duration Main Task	Acoustic Condition	Spatial Access	Success Criterion	Quantitative Metrics	Level Success Threshold
I. Environmental habituation	5 d; arena	None	Full	Voluntary exploration, absence of fear	1. Mean latency to enter any testing room 2. Frequency of fear-related (startle/flight, avoidance)	1. Mean latency ≤ 30 s (≥70% reduction from initial) 2. Fear ≤ 1 event h^−1^
II. Sound introduction	5 d; establishing sound safety association	Neutral ambient sound (30–40 dB)	Full	Calm during sound playback	1. Proportion of lying during sound playback 2. Frequency of aggressive	1. Lying proportion ≥ 30% 2. Aggression ≤ 2 events h^−1^
III. Music	7 d; acclimation to experimental music and volume	Experimental music (rotated), volume ramped (45 → 65 dB)	Full	Normal at target volume	1. Proportion of lying at target volume (65 dB) 2. Frequency of exploratory	1. Lying proportion ≥ 40% (achieved for 3 consecutive days) 2. Exploration ≥ 50 events h^−1^
IV. Protocol simulation	3 d; simulating full experimental procedure	Four rooms playing different experimental music	Full	Calm completion of choice procedure	1. Mean time to complete one full circuit of all testing rooms 2. Coefficient of variation (CV) of occupancy time per room	1. Mean exploration time ≤ 20 min 2. Occupancy time CV ≤ 0.3 (no strong spatial bias)

(1) Mean latency: time taken for a pig to move from the central activity zone into a testing room. (2) proportions: calculated as cumulative duration percentage within a 1 h observation window. (3) CV = standard deviation/mean, used to assess uniformity of spatial choice. (4) Acoustic specifications for habituation stages.

**Table 2:** Experimental conditions and sample sizes for behavioral data aggregation. Each pig (N = 72 total, tested in six cohorts of 12) had free access to all six testing rooms. The sample size listed for each condition represents the number of pigs for which behavioral data were collected in that specific acoustic environment.

Treatment Group	Description	Number of Pigs (*n*)	Primary Purpose
Control	No music/noise stimulus	12	Establish baseline; assess environmental effects.
Noise group	Non-musical pink noise	12	Control for generic sound exposure; isolate specific effects.
MF group	tempo Mozart (wind)	12	Test effect of tempo Western classical music.
MS group	tempo Mozart (wind)	12	Test effect of tempo Western classical music.
GF group	tempo *High Mountains and Flowing Water* (string)	12	Test effect of tempo Chinese classical music.
GS group	tempo *High Mountains and Flowing Water* (string)	12	Test effect of tempo Chinese classical music.
Total	6 groups	72	

**Table 3:** Correspondence between grained annotation labels and the aggregated categories used for analysis.

Category	Grained Annotation Labels	Rationale for Aggregation
Standing	standing, sitting	Both represent a non-locomotive, limb-supported posture indicative of alertness.
Walking	walk, run	Both entail self-propelled spatial displacement; ‘run’ is considered high-intensity walking. Combined to represent general activity.
Foraging	eat, nose-poke-elsewhere	‘Eat’ denotes direct feeding; ‘nose-poke-elsewhere’ includes rooting and sniffing exploratory components of foraging.
Drinking	drink	Direct correspondence.
Lying	lying, sleep	‘Sleep’ is a subset of lying (deep rest). Combined to represent total rest time.
Playing	with toys	Spontaneous, utilitarian activity indicative of positive welfare.
Exploring	investigating, nose-to-nose	‘Investigating’ is object or environment directed; ‘nose-to-nose’ is social investigation. Both reflect active information gathering.
Aggression	fight	Direct correspondence; denotes agonistic interaction.
Excretion	excretion	Direct correspondence; marks all defecation and urination events.

**Table 4:** Ethogram: definitions of recorded.

Type	Category	Operational Definition	Associated Affective Interpretation
State	Standing	Body supported by extended limbs with no visible displacement. The head may rotate or lower to investigate.	Relaxed
State	Walking	Purposeful locomotive movement resulting in a change body position, including forward, backward, or turning motion. At least one full gait cycle is completed.	Bored/ Curious
State	Foraging	Any sequence involving feeding- or drinking-related investigation. This includes: (1) Eating: muzzle inside the feeder with active chewing; (2) Rooting/Sniffing: head lowered, snout in contact with or disturbing the pen floor.	Content
State	Drinking	Continuous muzzle contact with the drinker, accompanied by swallowing motions, indicating active water intake. The bout starts at initial contact and ends upon withdrawal.	Focused
Event	Playing	Spontaneous, repetitive, and seemingly non-goal-directed locomotive activity, often exhibiting a relaxed bodily posture. Includes sudden bursts of running, jumping, or pivoting without an apparent external trigger, as well as object-directed play (e.g., nudging, chewing, or manipulating the suspended ball or wooden block) and social play (e.g., gentle chasing, nudging, or mounting of pen mates without agonistic escalation).	Lively/ Playful
State	Lying	Ventral body surface in contact with the ground in a resting posture. Subcategories: (1) Lateral lying: body on one side with limbs extended; (2) Ventral lying: sternum/abdomen on ground with limbs tucked underneath. Duration must exceed 3 s.	Sociable/ Content
Event	Exploring	Investigative oriented toward the environment or novel objects by slow, deliberate movement head raised ears forward and focused sniffing or visual inspection of a specific area or fixture (excluding the feeder). Distinguished from foraging by the absence of oral contact with feeder/waterer; distinguished from walking by the investigatory focus (sniffing, pausing) rather than directed locomotion.	Curious/Alert
Event	Excretion	A complete act of defecation or urination. Defecation: distinctive squatting posture with abdominal contraction and expulsion of. Urination: squatting (females) or standing (males) posture accompanied by urine flow.	Physiological
Event	Aggression	Agonistic or threatening interaction directed at a pen pal. Includes: (1) Pushing: forceful contact using the shoulder or head; (2) Biting: gripping any part of another pig’s body with the teeth (commonly ears or tail); (3) Chasing: persistent pursuit causing the recipient to flee. Distinguished from social play by the presence of high-pitched squeals, tense body posture, ears pinned back, and persistent targeting of a fleeing recipient. Play bouts, in contrast, involve reciprocal role reversals, relaxed body postures, and absence of distress vocalizations.	Aggressive/Fearful

(1) classified according to their temporal characteristics and mode of quantification. State (standing, walking, lying, foraging, drinking) are quantified as the percentage of total observation time. Event (excretion, playing, exploring, aggression) are quantified as frequency counts. (2) Representative still images are presented in the table. (3) The “Associated affective interpretation” column provides a general ethological context and is not derived from the QBA results of this study. It reflects common associations reported in the animal welfare literature and is included for illustrative purposes only.

**Table 5:** Two-way ANOVA of the effects of musical instrument type and tempo on pig.

Indicator	Instrument Type				Rhythm				Reciprocation	
	String Music	Wind Music	*F*(1, 188)	Value	Slow	Fast	*F*(1, 188)	Value	*F*(1, 188)	Value
	*n* = 24	*n* = 24					*n* = 24	*n* = 24		
State (%) Standing	4.2 ±1.5	4.0 ±1.4	0.3	0.581	3.5 ^b^ ±1.2	4.7 ^a^ ±1.6	12.5	<0.001	1.2	0.285
Walking	6.8 ±2.3	7.5 ±2.8	1.5	0.223	5.9 ^b^ ±2.0	8.4 ^a^ ±2.6	25.8	<0.001	10.5	0.002
Lying	45.6 ±8.7	42.1 ±9.2	3.2	0.079	48.3 ^a^ ±7.5	39.4 ^b^ ±9.0	24.1	<0.001	18.7	<0.001
Drinking	5.6 ±1.8	4.9 ±1.7	4.2	0.044	5.8 ^a^ ±1.6	4.7 ^b^ ±1.8	9.7	0.003	6.4	0.014
Foraging	5.8 ±1.9	5.2 ±1.7	2.5	0.117	6.1 ^a^ ±1.8	4.9 ^b^ ±1.6	9.8	0.003	0.7	0.407
Event (N) Excretion	1.8 ±0.7	2.1 ±0.8	3.9	0.052	1.7 ^b^ ±0.6	2.2 ^a^ ±0.8	11.2	0.001	0.1	0.752
Playing	15.7 ±5.2	12.4 ±4.8	8.1	0.006	13.8 ±4.9	14.3 ±5.5	0.2	0.670	5.0	0.029
Exploration	120.5 ±25.4	105.3 ±30.1	5.8	0.019	125.8 ^a^ ±22.7	98.7 ^b^ ±29.0	19.4	<0.001	2.3	0.135
Aggression	2.1 ±1.0	2.4 ±1.2	1.4	0.245	1.8 ^b^ ±0.9	2.7 ^a^ ±1.1	15.9	<0.001	0.8	0.385
Preference indicator(s) Occupancy time	285 ±45	260 ±52	5.0	0.029	275 ±48	270 ±51	0.2	0.654	62.3	<0.001

(1) Values represent mean ± SD. Within a given main-effect column (e.g., Tempo), different superscript letters (a, b) indicate statistically significant differences at *p* < *0.05.* Interpretation of interactions: A significant interaction term (*p* < *0.05*) indicates that the effect of one acoustic parameter depends on the level of the other. For instance: The significant interaction for walking signifies that the increase in walking induced by a fast tempo was stronger in MF stimulus condition than in GS stimulus condition. The highly significant interactions for lying and occupancy time demonstrate that the promoting effect of a slow tempo on these measures was greatest when paired with the GS stimulus, defining the optimal GS combination. (2) For the two-way ANOVA, the sample size for each main effect level (e.g., String, Fast) is *n* = 24. This value represents the pooled data from the two corresponding treatment groups, each n=12, within the 2 × 2 factorial design. The total number of pigs included in this analysis is N = 48. (3) Note that the two-way ANOVA treats observations across instrument and tempo conditions as independent. The free-choice design implies repeated measures, and the reported *p*-values should be interpreted with this analytical limitation in mind.

**Table 6:** One-way ANOVA and Tukey HSD post-hoc test table.

Indicator Classification	Index	GS (*n* = 12)	GF (*n* = 12)	MS (*n* = 12)	MF (*n* = 12)	C (*n* = 12)	N (*n* = 12)	F (5, 66)	Value	Tukey HSD (α = 0.05)
State (%)	Standing	3.8 ±0.4	4.5 ±0.5	3.9 ±0.4	4.8 ±0.6	4.1 ±0.5	4.3 ±0.5	2.31	0.053	n.s.
	Walking	6.0 ±0.7 ^b^	7.6 ±0.9 ^a^	5.8 ±0.6 ^b^	9.1 ±1.1 ^a^	6.2 ±0.7 ^b^	6.5 ±0.8 ^b^	8.95	<0.001	MF, GF > GS, MS, C, N
	Lying	53.2 ±4.5 ^a^	38.5 ±3.8 ^c^	46.8 ±4.1 ^b^	35.9 ±3.5 ^c^	44.7 ±4.0 ^b^	41.3 ±3.9 ^bc^	15.47	<0.001	GS > MS, C > GF, MF, N
	Drinking	6.2 ±0.6 ^a^	4.8 ±0.5 ^b^	5.1 ±0.5 ^b^	4.6 ±0.5 ^b^	5.0 ±0.5 ^b^	4.5 ±0.5 ^b^	7.21	<0.001	GS > GF, MF, MS, C, N
	Foraging	6.5 ±0.7 ^a^	5.0 ±0.6 ^b^	6.0 ±0.6 ^ab^	4.8 ±0.5 ^b^	5.8 ±0.6 ^ab^	5.1 ±0.6 ^b^	5.88	<0.001	GS > GF, MF, N
	Playing	14.2 ±2.1 ^b^	12.8 ±1.9 ^b^	11.5 ±1.7 ^b^	19.5 ±2.8 ^a^	10.5 ±1.6 ^b^	7.8 ±1.2 ^c^	18.34	<0.001	MF > (GS, GF, MS, C) > N
Event (N)	Exploration	132.5 ±15.8 ^a^	108.3 ±13.2 ^b^	119.0 ±14.5 ^ab^	95.2 ±11.6 ^c^	115.8 ±14.0 ^b^	89.5 ±10.9 ^c^	12.63	<0.001	GS > (GF, C, MS) > (MF, N)
	Aggression	1.5 ±0.3 ^c^	2.3 ±0.4 ^ab^	1.8 ±0.3 ^bc^	2.8 ±0.5 ^a^	1.9 ±0.4 ^bc^	3.2 ±0.6 ^a^	16.89	<0.001	(N, MF) > (C, MS, GF) > GS
	Excretion	1.6 ±0.3 ^b^	1.9 ±0.4 ^ab^	1.7 ±0.3 ^b^	2.3 ±0.5 ^a^	1.8 ±0.4 ^b^	2.1 ±0.4 ^a^	4.05	0.003	(MF, N) > (GS, MS, C)
	Ear and tail lesion rate(%)	0.5 ±0.2 ^c^	1.8 ±0.5 ^b^	0.8 ±0.3 ^c^	3.2 ±0.8 ^a^	1.2 ±0.4 ^b^	4.1 ±1.0 ^a^	25.76	<0.001	(N, MF) > (GF, C) > (MS, GS)
Preference indicator	Occupancy time (s)	335 ±38 ^a^	268 ±32 ^b^	247 ±29 ^bc^	292 ±35 ^b^	251 ±30 ^bc^	182 ±25 ^c^	25.18	<0.001	GS > (GF, MF) > (MS, C) > N

(1) Data format: Values are mean ± SD. State (standing, walking, lying, drinking, foraging) are expressed as percentage of observation time. Event (excretion, playing, exploring, aggression) are total frequencies (n). Occupancy time is in seconds (s). Post hoc test (Tukey HSD): Superscript letters (a, b, c) denote homogenous subgroups. Groups sharing a letter do not differ significantly (*p* > 0.05); different letters indicate significant differences (*p* < 0.05). Letters are ordered (a > b > c) by descending mean value. Significance notation: *p* < 0.001 is reported as highly significant; *p* < 0.05 as significant; “n.s.” indicates non-significance. Lesion rate: The ear/tail lesion rate is defined as the percentage of pigs per group with a lesion score ≥ 1 during observation. This metric is a validated indicator of chronic stress and welfare status in pigs. (2) One-way ANOVA was used for simplicity. The repeated-measures nature of the free-choice design is acknowledged in Section 4.6. The large effect sizes observed for key comparisons support the biological robustness of the findings. (3) Sample size and experimental unit. Each condition in this table represents data aggregated at the individual pig level from all 72 pigs (tested in six cohorts of 12) that had free access to all six rooms. Thus, *n* = 72 for each condition. One-way ANOVA treats these observations as independent across conditions; the repeated-measures nature of the design is acknowledged in the Limitations (Section 4.6).

**Table 7:** ACTH concentrations across treatment groups.

Treatment Group	Serum Cortisol (nmol L^−1^)	Serum ACTH (pg mL^−1^)
GS (slow-tempo GS stimulus)	25.3 ± 3.1 ^c^	15.8 ± 2.4 ^c^
MF (fast-tempo MF stimulus)	31.5 ± 4.2 ^b^	20.1 ± 3.0 ^b^
Silent control (C)	34.8 ± 4.0 ^b^	22.5 ± 3.3 ^b^
Noise control (N)	48.7 ± 5.6 ^a^	32.5 ± 4.1 ^a^
Statistical significance	*F(3, 28)* = 35.2, *p < 0.001*	*F(3, 28)* = 28.7, *p < 0.001*

Different superscript letters within a column indicate significant differences (Tukey’s HSD, *p < 0.05*).

**Table 8:** PCA loadings of QBA descriptors on the first two principal components.

Descriptor	PC1 Loading (53.2%)	PC2 Loading (25.3%)
Relaxed	**0.88**	−0.12
Content	**0.79**	0.08
Sociable	**0.72**	0.31
Calm	**0.85**	−0.22
Aggressive	**−0.76**	0.18
Fearful	**−0.69**	−0.05
Frustrated	**−0.64**	0.27
Lively	0.15	**0.82**
Playful	0.22	**0.78**
Curious	0.35	**0.69**
Alert	−0.08	**0.61**
Focused	0.42	0.48
Bored	−0.51	−0.56

Note: Loadings >0.60 or <−0.60 are highlighted in bold.

## Data Availability

The datasets generated and/or analyzed during the current study are available from the corresponding author on reasonable request. The code used for the automated recognition system is available at https://github.com/wzj-jpg/YOLOv11 (accessed on 2 May 2026).

## Supplementary Materials

The following supporting information can be downloaded at: https://www.mdpi.com/article/10.3390/ani16101475/s1, Figure S1. Network architecture diagram of YOLOv11; Figure S2. Working principle diagram of the Adaptive Spatial Feature Fusion (ASFF); Figure S3. Experimental findings and confusion matrix of ASFF_YOLOv11; Figure S4. Network architecture diagram of FASFFHead; Figure S5. a. Precision-Recall (PR) curve and confusion matrix for the P2 layer of Detect_FASFF_YOLOv11; b. Precision-Recall (PR) curve and confusion matrix for the P6 layer of Detect_FASFF_YOLOv11; Figure S6. Schematic architecture of the improved YOLOv11 model with the Hierarchical Feature Fusion Block (HFFB); Figure S7. Overall architecture of the HiFuse model; Figure S8. Detailed architecture of the High-Frequency Fusion (HFF) module; Figure S9. Experimental findings of HFPN_YOLOv11; Figure S10. Comparison of mAP50, mAP50–95, recall and precision metrics; Figure S11. Comparison of loss functions; Figure S12. Confidence intervals using 423,407 and 2026 random seeds; Figure S13. Comparison between the Fine Analysis Diagram and the Model Diagram; Table S1. Ablation of the FASFF group; Table S2. HFPN_YOLOv11; Table S3. Table; Table S4. Comparison of Average Precision (AP) among.

## Abbreviations

The following abbreviations are used in this manuscript:

ACTH	Adrenocorticotropic hormone
ANOVA	Analysis of variance
ASFF	Adaptive Spatial Feature Fusion
bpm	Beats per minute
C	Silent control group
CV	Coefficient of variation
ELISA	Enzyme-linked immunosorbent assay
F	F-statistic
FASFF	Feature-adaptive Spatial Feature Fusion
fps	Frames per second
G	*High Mountains and Flowing Water* (GS stimulus)
GF	Fast-tempo GS stimulus group
GS	Slow-tempo GS stimulus group
HFF	High-Frequency Fusion
HFFB	Hierarchical Feature Fusion Block
HFPN	Hierarchical Feature Pyramid Network
HPA axis	Hypothalamic pituitary adrenal axis
HSD test	Honestly Significant Difference test
ICC	Intraclass correlation coefficient
lx	Lux
M	Mozart’s Piano Concerto No. 23 (MF stimulus)
mAP	Mean average precision
mAP_50_	Mean average precision at 0.5 IoU threshold
MF	Fast-tempo MF stimulus group
MS	Slow-tempo MF stimulus group
N	Noise control group
n.s.	Not significant
QBA	Qualitative Assessment
SD	Standard deviation
SGD	Stochastic gradient descent
SPL	Sound pressure level
SPSS	Statistical Package for the Social Sciences
YOLO	You Only Look Once (object detection algorithm)
Symbols	
d	Cohen’s effect size
F	Variance ratio in ANOVA
Hz	Hertz (s-1)
IoU	Intersection over Union threshold
lx	Lux (illuminance)
N	Sample size (number of animals)
n	Subgroup sample size
R^2^	Coefficient of determination
s	Second
t	Time
κ	Cohen’s kappa coefficient
Greek symbols	
α	Significance level (Type I error rate)
β	Probability of Type II error
κ	Cohen’s kappa coefficient (inter-observer reliability)
μ	Population mean
ε	Residual error
θ	Rotation angle (degrees)
