# Supplementary material for: Influence of Specific Acoustic Parameters on Responses in Growing Pigs: Towards a Precision Auditory Enrichment Strategy
Source: Animals (Basel). 2026 May 11;16(10):1475. doi: 10.3390/ani16101475 (PMC13203548; doi:10.3390/ani16101475)
Supplement: Supplementary file 1 [file animals-16-01475-s001.zip › animals-4294188-supplementary.pdf]

Influence of Specific Acoustic Parameters on Behavioral Responses in Finishing Pigs: Towards a Precision Auditory Enrichment Strategy

Model Description and Experimental Results and Analysis of YOLO

To enable high-throughput, objective analysis of pig behavior within the free-choice paradigm, this study employed the YOLOv11 (You Only Look Once version 11) architecture. As a state-of-the-art single-stage object detector, YOLOv11 is engineered to achieve an optimal balance between inference speed and detection accuracy, a prerequisite for processing continuous video streams in real-time applications.

YOLOv11 incorporates systematic enhancements over its predecessors, including a reinforced backbone network for robust feature extraction, an advanced feature pyramid network (neck) for multi-scale feature fusion, and an adaptive detection head. These refinements collectively improve the model's capability for precise and efficient object detection in dynamic environments. Consequently, YOLOv11 is particularly suited for automated, continuous monitoring scenarios such as tracking and identifying nuanced animal behaviors in farm settings where both temporal resolution and analytical precision are critical.

In this work, we implemented the YOLOv11n (nano) configuration. This variant was selected based on its alignment with the computational constraints of our research platform, ensuring operational feasibility without compromising the requisite detection performance for behavioral phenotype. The model follows a proven multi-stage architecture (**Figure S1**), where its integrated modules operate cohesively to maintain high accuracy while minimizing computational overhead.

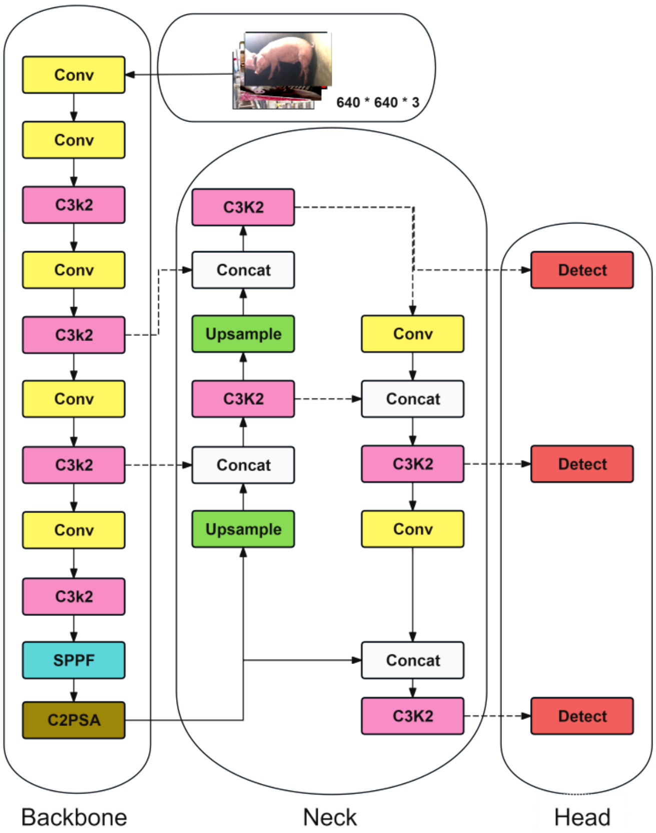

Figure S1. Network architecture diagram of YOLOv11.

Detect_FASFF_YOLOv11

Methodological Enhancement: Adaptive Spatial Feature Fusion (ASFF)

A persistent challenge in object detection is the effective handling of scale variation. While feature pyramid networks are commonly employed to address this, single-shot detectors utilizing such architectures often exhibit inconsistencies across different feature scales, limiting their detection robustness. To resolve this, we introduced an Adaptive Spatial Feature Fusion (ASFF) module into our YOLOv11-based framework.

The principal innovation of ASFF is a learnable, spatial weighting mechanism that dynamically fuses multi-scale feature maps. By deceptively calibrating the contribution of each pyramid level at every spatial location, ASFF selectively enhances coherent features

while suppressing conflicting information across scales. This significantly improves the model's scale-invariant representational capacity.

Applied to our porcine behavior recognition task, the integration of ASFF led to a marked performance improvement. The mean average precision at a 0.5 Intersection-over-Union threshold (mAP_{50}) increased by 8.5%, with particularly pronounced gains in detecting subtle, small-scale behaviors such as aggressive interactions.

Notably, the ASFF module achieves these enhancements with minimal computational overhead, preserving the efficiency essential for real-time, continuous behavioral monitoring. The architecture and performance outcomes of the ASFF module are detailed in **Figure S2** and **S3**, respectively.

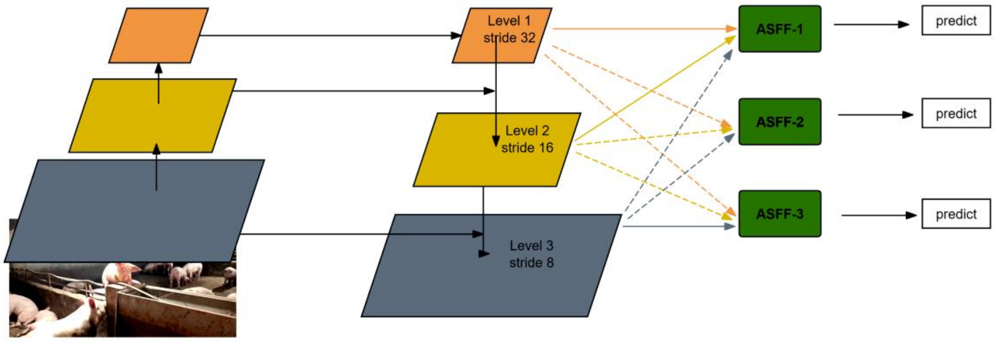

Figure S2. Working principle diagram of the Adaptive Spatial Feature Fusion (ASFF). Level 1, Level 2, and Level 3 signify the feature levels within the feature pyramid, each characterized by distinct spatial resolutions. ASFF-1, ASFF-2, and ASFF-3 represent feature fusion at different levels where the ASFF mechanism is implemented.

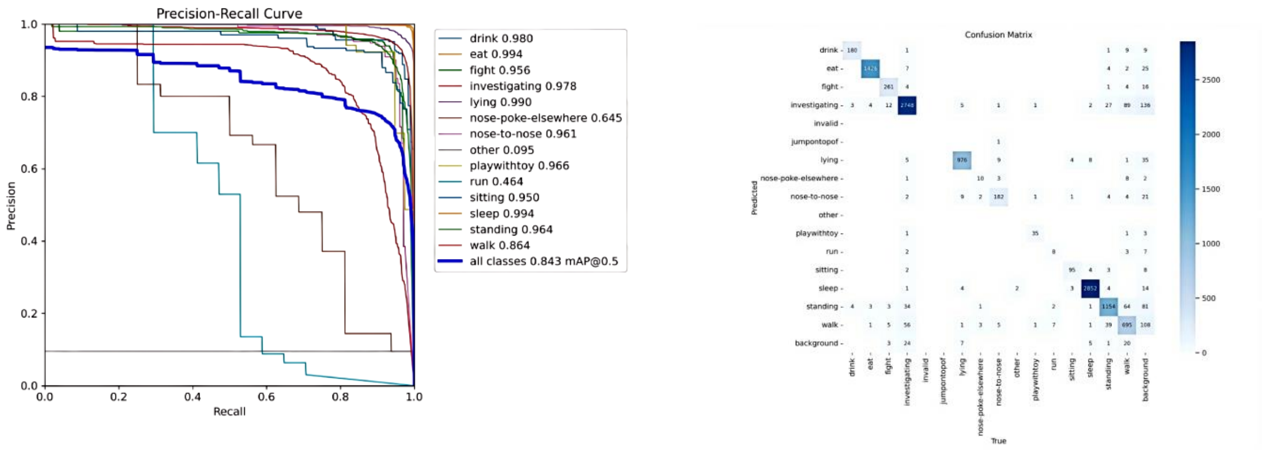

Figure S3. Experimental findings and confusion matrix of ASFF_YOLOv11.

Architectural Innovation: Feature-Augmented Spatial Feature Fusion Head

While the Adaptive Spatial Feature Fusion (ASFF) mechanism provides a foundation for multi-scale feature integration, its conventional formulation exhibits a key limitation. It computes spatial weights primarily from features within a single layer, failing to leverage broader contextual relationships across different network levels or to model interactions along the channel dimension. This can constrain the discriminate quality of the fusion weights, particularly in complex farm environments characterized by high animal density, intermittent occlusion, or pronounced scale variation among behavioral targets.

To overcome this constraint, we developed an enhanced detection head termed the Feature-Augmented Spatial Feature Fusion Head (FASFFHead). This innovation introduces a dual-path feature enhancement module designed to preserve the adaptive spatial selection strength of ASFF while substantially augmenting the representational capacity of the weight generation process. The FASFFHead explicitly models cross-layer dependencies and channel-wise interactions, thereby mitigating feature degradation during multi-scale fusion.

Furthermore, it incorporates a secondary feature refinement stage specifically optimized for detection layers responsible for both large and small targets.

The proposed FASFFHead architecture is detailed in **Figure S4**, illustrating its integration into the overall detection pipeline and its role in advancing robust behavioral recognition under challenging conditions.

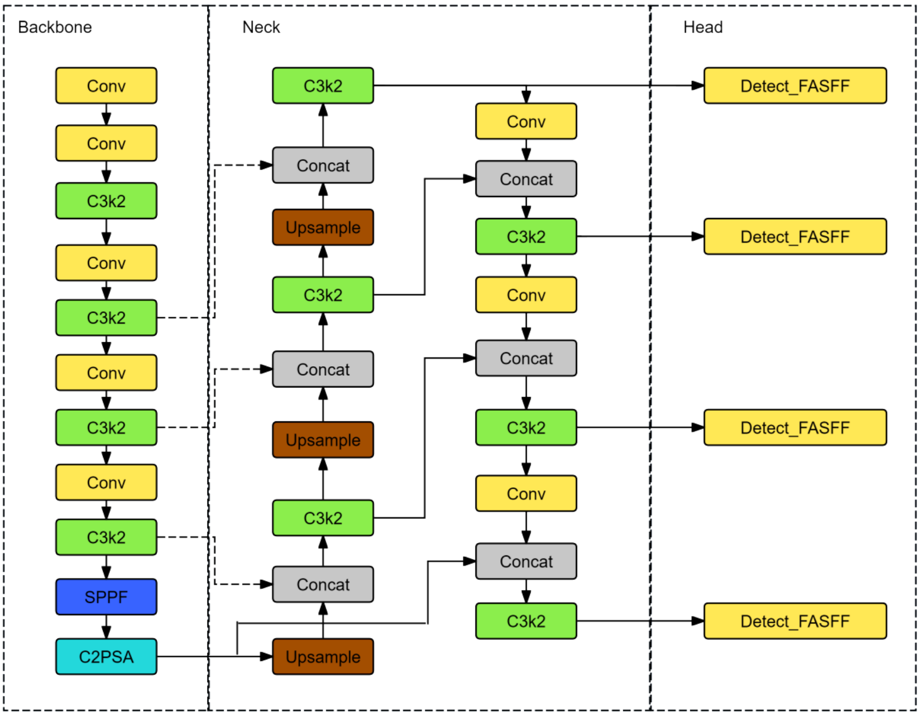

Figure S4. Network architecture diagram of FASFFHead.

The proposed FASFFHead introduces a dual-path weight generation mechanism as its core innovation. This architecture is designed to compute spatially adaptive fusion weights by simultaneously incorporating fine-grained local details and semantically rich global context, thereby producing more discriminate and robust weighting maps.

Regarding the feature map of the L-th layer that necessitates fusion$x_l \in R^{H \times W \times C}$, in this research, the enhanced weight tensor was produced via the following procedures:

1. Local Detail Path: This branch preserves high-resolution spatial information. The input

feature x_l is processed by a lightweight constitutional sequence, a 3×3 depth-wise separable convolution, followed by a 1×1 convolution to extract detailed local features, denoted as F_{local}.

2. Global Context Path: To capture scene-level semantics, this branch first applies Global Average Pooling (GAP) to x_l to produce channel-wise statistics. These statistics are then transformed by a compact Multi-Layer Perceptron (MLP) containing two fully connected layers with nonlinear activation. The resulting context vector is spatially broadcast and added element-wise back to x_l, yielding the globally informed feature representation F_{global}.

The outputs from both paths are concatenated to form a composite feature representation:

$$F_{fused}=Concat(F_{local}, F_{global}) \quad (1)$$

This combined feature F_{fused} is then passed through a 1×1 constitutional layer for dimensional reduction and integration, followed by a Soft max normalization across the level dimension to produce the final spatially adaptive weight map α_l.

The subsequent fusion step follows the standard ASFF formulation. All multi-scale features are first transformed to a common spatial resolution and then aggregated via the computed adaptive weights:

$$y=\sum_{l=1}^{N} \alpha_l \cdot Resize\ (x_l, target\_shape) \quad (2)$$

where the summation runs over the N detection heads (a three-head configuration was adopted in this study). Conductress sampling or down-sampling operations are applied to standardize all feature maps.

To rigorously evaluate the contribution of each component within the FASFF module, a systematic ablation study was performed using the standard YOLOv11n model as the baseline. The effectiveness of the proposed enhancements was quantified by the improvement

in the mean Average Precision at a 0.5 Intersection over Union (mAP_{50}), while computational efficiency was assessed via Floating Point Operations (FLOPs). The complete results of this component-wise analysis are presented in **Table S1**.

Table S1. Ablation study of the FASFF group.

Model Variant	Spatial Alignment	Co-Attention	mAP $_{50}$ (%)	FLOPs (G)	mAP_{50-95} (%)
Baseline (YOLOv11n)	-	-	75.8%	21.3	62.9%
FASFF w/Naive Fusion	√	-	90.5% (+14.7%)	8.9	78.6% (+15.7%)
FASFF w/Alignment Only	-	√	90.7% (+14.9%)	9.3	78.9% (+16.0%)
FASFF (Full)	√	√	91.9% (+16.1%) 91.5% (+15.7%)	8.4 8.1	81.5% (+18.6%) 80.8% (+17.9%)

Note: The symbol "–" denotes the absence of the feature; "√" denotes the presence of the feature.

Performance Evaluation: Ablation Study and Final Model Results

The component-wise ablation study clarifies the contribution of each element within the proposed FASFFHead architecture. Initial feature integration via simple element-wise addition provided a foundational improvement of 14.7% in mean Average Precision (mAP_{50}), confirming the value of multi-scale feature combination. Implementing spatial alignment alone yielded a significant performance gain of 14.9% mAP_{50}, highlighting its essential role in resolving scale inconsistencies. The complete FASFF module, incorporating its cooperative attention mechanism for adaptive weighting, achieved the highest improvement of 15.9% ±

0.2% mAP_{50}. This progression demonstrates the critical importance of spatially adaptive, context-aware weighting for effective multi-scale feature fusion in complex behavioral recognition scenarios.

Following 100 epochs of training, the enhanced Detect_FASFF_YOLOv11 model demons trated state-of-the-art performance in automated porcine behavior recognition. On the h eld-out test set, it achieved a mean Average Precision at a 0.5 Intersection over Union (mAP_{50}) of 91.7%, a substantial increase of 16 percentage points over the baseline YOL Ov11n model.

This marked improvement is directly attributable to the dual-path weight generation mechanism of the FASFFHead. By simultaneously modeling local spatial details and global semantic context, the module overcomes the limited discriminative capacity of conventional ASFF in challenging farm environments characterized by occlusion and scale variation. Notably, the model showed particular strength in detecting small-scale behaviors; for instance, recognition accuracy for subtle aggressive interactions improved by 3.2%, underscoring the module's efficacy in preserving and enhancing fine-grained features. These results comprehensively validate the proposed architectural innovations, confirming their effectiveness and practical relevance for high-precision, automated behavior monitoring in commercial pig production. A summary of the experimental outcomes is presented in **Figure S5**.

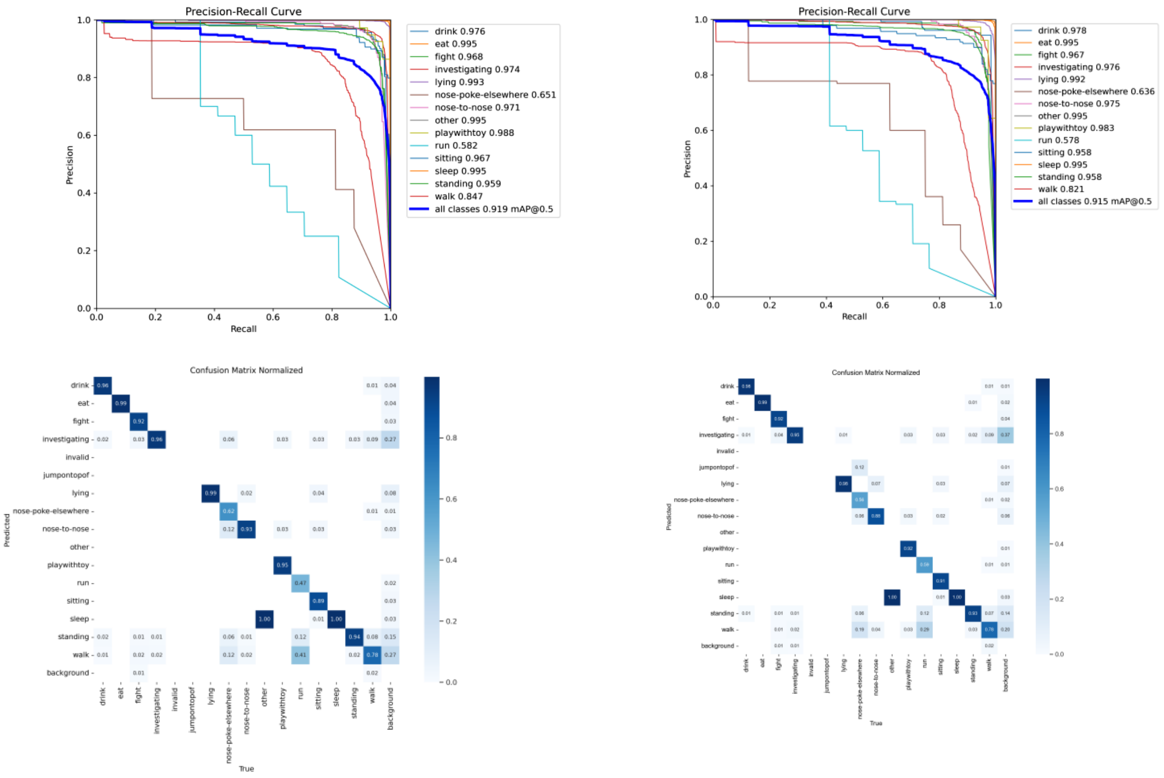

(a) **(b)**

Figure S5. a. Precision-Recall (PR) curve and confusion matrix for the P2 layer of Detect_FASFF_YOLOv11; **b**. Precision-Recall (PR) curve and confusion matrix for the P6 layer of Detect_FASFF_YOLOv11.

HFPN_YOLOv11

Accurate recognition of intricate, multi-scale behaviors in group-housed finishing pigs requires a feature representation that robustly integrates both fine-grained spatial details and higher-level semantic context. While conventional Feature Pyramid Networks (FPNs) combine multi-scale features through top–down addition or concatenation, their relatively simple fusion mechanisms offer limited capacity to model complex intra- and inter-scale dependencies. This can constrain performance when analyzing pig behavior, a domain characterized by large posture variations, significant scale differences (e.g., between 'lying' and 'nose-to-nose interaction'), and frequent partial occlusion.

To address this challenge, we introduce an enhanced neck architecture termed the Hierarchical Feature Pyramid Network (HFPN), a core methodological contribution of this work. The HFPN incorporates a novel Hierarchical Feature Fusion Block (HFFB) designed to facilitate more refined and context-aware integration across feature scales. The complete HFPN structure is detailed in **Figure S6**.

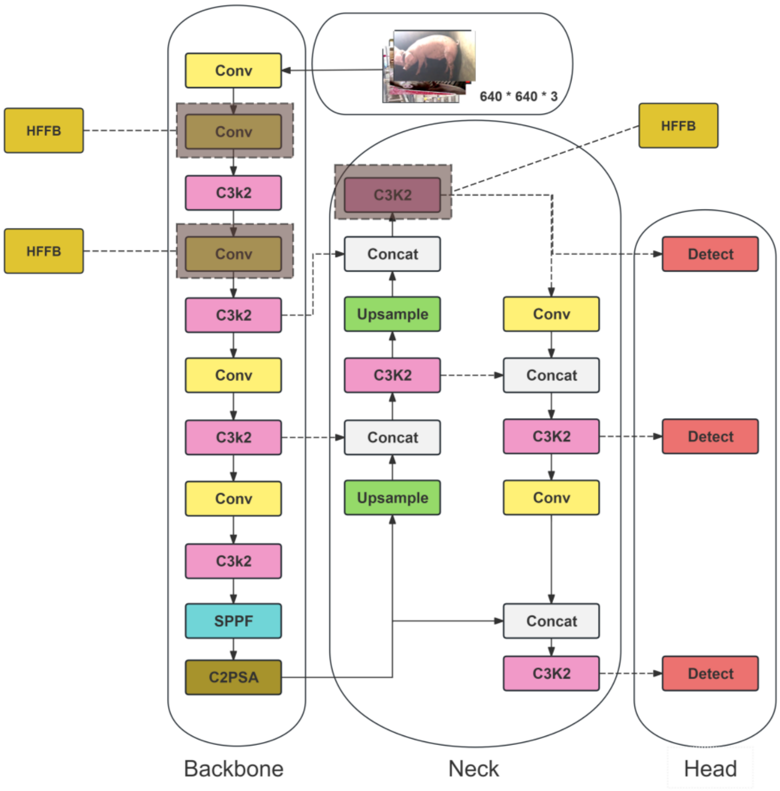

Figure S6. Schematic architecture of the improved YOLOv11 model with the Hierarchical Feature Fusion Block (HFFB).

The backbone network extracts multi-scale features (P3, P4, P5). The proposed HFFB module (highlighted in blue) adaptively learns channel-wise weights (α, β, γ) to perform a weighted fusion of these features, giving prominence to detailed (P3) or semantic (P5) information based on the input. This customized design, particularly focusing on the P3 level fusion path

as configured in our study, significantly enhances the model's capability to discern subtle and small target pig behaviors (e.g., nose-to-nose, investigating) from our 14 category fine-grained dataset, enabling precise behavioral analysis for welfare assessment.

Design and Function of the Hierarchical Feature Fusion Block

The Hierarchical Feature Fusion Block (HFFB) is designed to enable a more refined and context-aware integration of multi-scale features from the backbone network. As illustrated in **Figure S7** and **Figure S8**, the HFFB processes feature maps from three distinct hierarchical levels, each contributing complementary information essential for robust behavior recognition:

High-Level Feature Map (P5)

This map encapsulates rich global semantic context, such as the overall posture of a 'play' sequence or spatial relationships within a group of pigs. However, its representation lacks fine-grained spatial detail.

Mid-Level Feature Map (P4)

Offering an optimal balance between semantic meaning and spatial resolution, this level provides critical information for recognizing common, posture-defined behaviors like 'walking' or 'exploring'.

Low-Level Feature Map (P3)

Retaining high-resolution local details and edge information, this map is vital for detecting subtle anatomical features (e.g., ear or tail orientation) and precise contact points, such as in 'nose-to-nose' interactions.

By explicitly modeling and fusing these hierarchically distinct representations, the HFFB

constructs a composite feature space that simultaneously captures broad behavioral context and fine-grained postural details, thereby providing the subsequent detection head with a more discriminative foundation for accurate classification.

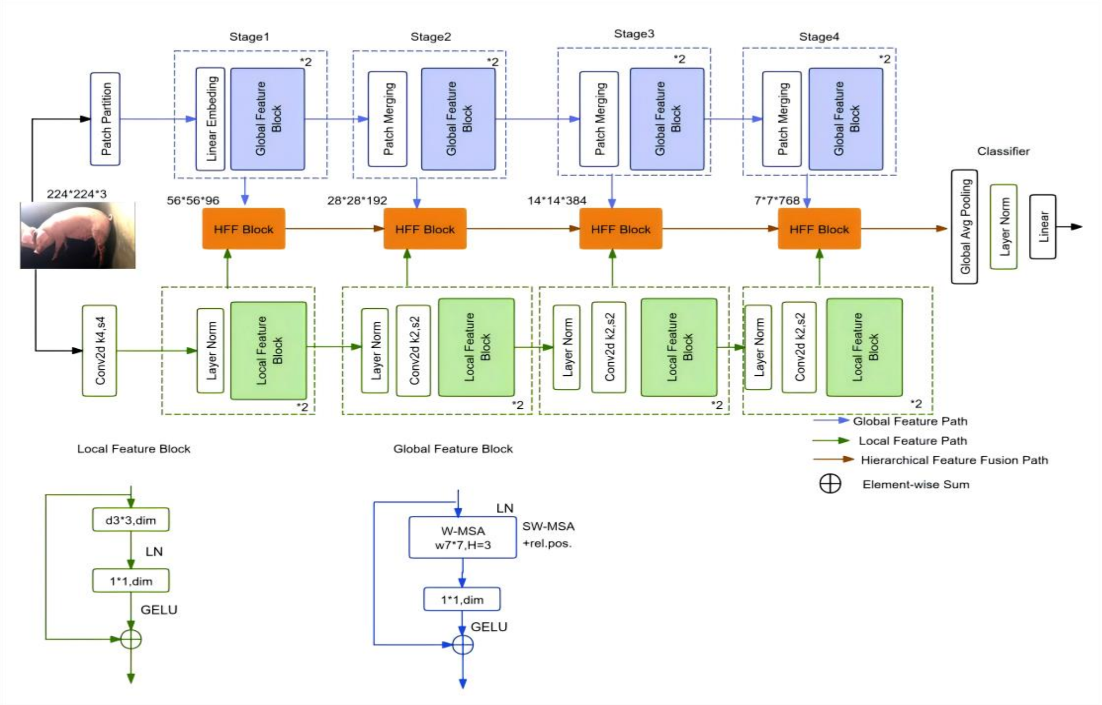

Figure S7. Overall architecture of the HiFuse model.

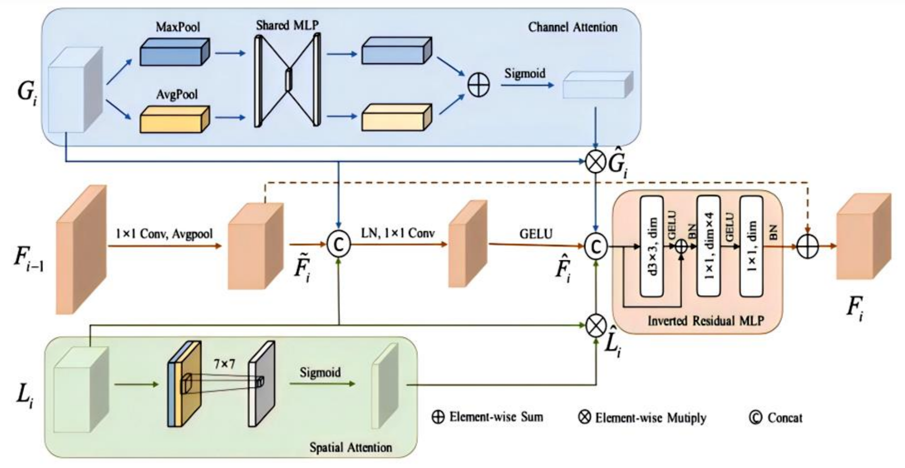

Figure S8. Detailed architecture of the High-Frequency Fusion (HFF) module.

Operational Mechanism of the Hierarchical Feature Fusion (HFF) Module

The HFF module at each stage integrates three complementary inputs to construct a rich,

multi-faceted feature representation: (1) Global feature G_i : encodes overarching scene context and inter-animal relationships; (2) Local feature L_i: retains fine-grained spatial details of individual posture and anatomy; (3) Cross-scale feature $F_{i\text{-}1}$: propagated from the preceding stage, preserving refined information from other network levels. The module's objective is to adaptively refine and fuse these inputs through a structured sequence of attention and transformation operations.

Step 1: Dual-Attention Feature Refinement

Global Pathway (Channel Attention): To emphasize the most informative semantic channels, G_i undergoes parallel average pooling and max pooling. The resulting vectors are processed by a shared Multi-Layer Perceptron (MLP) to model channel-wise dependencies. A sigmoid-activated attention map is generated and applied via element-wise multiplication:

$$\widehat{G}_i=\sigma(MLP\ (AvgPool\ (G_i))+MLP\ (MaxPool\ (G_i)))\odot G_i. \quad (3)$$

In this context, σ represents the Sigmoid function, and $\odot$ denotes element-wise multiplication, weighted global feature $\widehat{G}_i$.

Local Pathway (Spatial Attention): To focus on behaviorally salient regions within L_i, a 7×7 convolutional filter chosen to match the spatial scale of key postural cues (e.g., head orientation) is applied. The output is activated by a sigmoid function to produce a spatial attention mask:

$$\hat{L}i=\sigma(f7\times 7\ (L_i))\odot L_i \quad (4)$$

Step 2: Cross-Scale Hierarchical Fusion

The refined global feature G_i is first concatenated with the historical cross-scale feature $F_{i\text{-}1}$

to incorporate broader contextual information. This combined representation is normalized via Layer Normalization (LN), then integrated through a 1×1 convolution and a GELU activation function. The resulting tensor is subsequently fused with the attended local feature $\hat{L}i$ through element-wise multiplication, enabling an initial synthesis of global context and localized detail.

Step 3: Non-Linear Feature Enhancement

To significantly boost the representational capacity of the fused features, a lightweight Inverted Residual MLP block is employed. This block follows a depth-wise separable design: Depth-wise Convolution (channel reduction) → GELU → Batch Normalization → 1×1 Convolution (expansion) → GELU → 1×1 Convolution (reduction) → Batch Normalization, enhanced with a residual connection. This structure efficiently models complex, non-linear behavioral patterns—such as distinguishing between 'investigating' and 'foraging'—with minimal computational overhead.

In essence, the HFF module implements a cohesive pipeline: channel-wise attention filters global semantics, spatial attention highlights local key regions, cross-scale fusion enriches contextual detail, and an inverted residual MLP strengthens non-linear representational power. This integrated design directly addresses a fundamental limitation in video-based behavior analysis: the denationalization of multi-scale feature relationships by enabling the model to energetically leverage both macroscopic scene cues and microscopic postural details. The resulting enhanced architecture, designated HFPN_YOLOv11, provides the robust, high-resolution feature foundation necessary for the accurate classification and localization of 14 distinct pig behaviors. This technical advancement is instrumental in

generating the precise behavioral metrics required to rigorously evaluate the efficacy of our precision auditory enrichment strategy.

Experimental Validation of the HFFB Module

To rigorously evaluate the effectiveness of the proposed Hierarchical Feature Fusion Block (HFFB), a controlled ablation study was performed. The study utilized our proprietary data set, annotated in detail for 14 distinct pig behaviors, with all models trained for 100 epochs. The results of this component-wise analysis are summarized in **S9**.

Furthermore, a direct performance comparison was conducted between the baseline YOLOv11n model and our novel HFPN_YOLOv11 architecture, which integrates the HFFB. The models were assessed using the standard object detection metric mean Average Precision at a 0.5 Intersection over Union threshold (mAP$_{50}$). The comparative results are presented in **Table S2**.

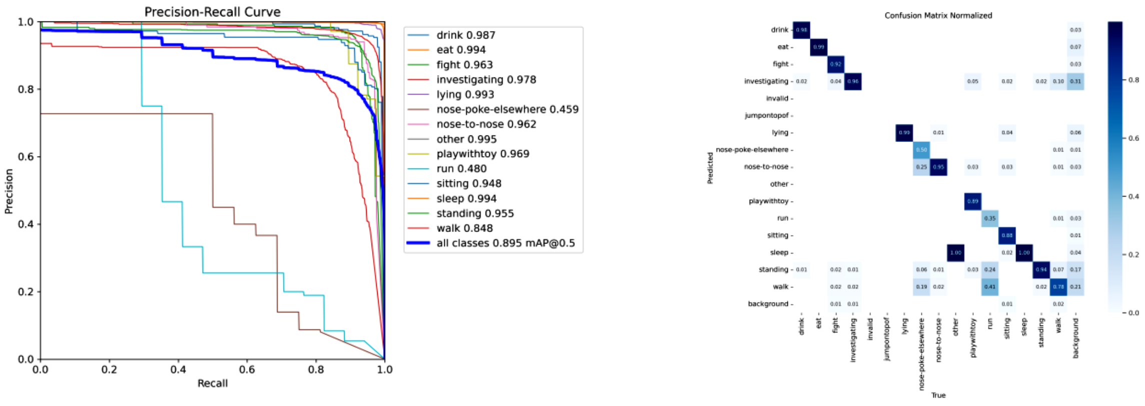

Figure S9. Experimental findings of HFPN_YOLOv11.

Table S2. HFPN_YOLOv11 ablation experiment.

Model	mAP$_{50}$ (%)	FLOPs (G)	Params (M)	mAP$_{50-95}$ (%)
YOLOv11n	75.8%	9.4	21.3	62.9%
HFPN_YOLOv11	89.5%	13.5	11.5	78.1%

Results demonstrate that the enhanced model achieves a significant improvement in overall detection accuracy (mAP_{50}), with particularly pronounced gains in recognizing small-scale behavioral categories such as exploration and social interaction. This performance enhancement is attained with only a marginal increase in computational cost.

These outcomes provide direct validation for the efficacy of the HFFB module. By refining the multi-scale feature-integration process, the module substantively enhances the model's discriminate capacity within the complex repertoire of porcine behaviors. This architectural advancement thereby establishes a robust and precise technical foundation essential for the subsequent high-resolution behavioral quantification central to this investigation.

Result

Performance Comparison with Other YOLO Series Models

The enhanced YOLOv11 architecture was employed to automatically identify and quantify nine key pig behaviors across the six experimental acoustic conditions. In the complex task of automated animal behavior recognition, it is critical to rigorously evaluate whether model improvements translate into measurable performance gains. We therefore conducted a controlled comparative experiment designed to directly validate the effectiveness, superiority, and robustness of the proposed modifications.

Our evaluation framework extends beyond achieving high scores during training; it prioritizes the model's stability and practical reliability, especially in challenging scenarios such as identifying subtle, stress-induced behavioral cues under variable farm conditions. All models in this comparison were trained from scratch without leveraging ore-trained weights to ensure a fair assessment of architectural differences. The baseline YOLOv11 model was

compared against its enhanced variants using standard object detection metrics, including mean Average Precision at a 0.5 Intersection over Union (mAP@0.5), mAP across IoU thresholds from 0.5 to 0.95 (mAP@0.5:0.95), recall, and training loss. The results demonstrate consistent and significant improvements across these metrics, underscoring the model's potential for reliable health and welfare monitoring in smart farming applications. The precise and timely detection of behaviorally expressed health indicators is vital for early intervention. Furthermore, the model's design ensures computational efficiency suitable for real-world deployment on farms. A detailed presentation of these comparative results is provided in **Figure S10**.

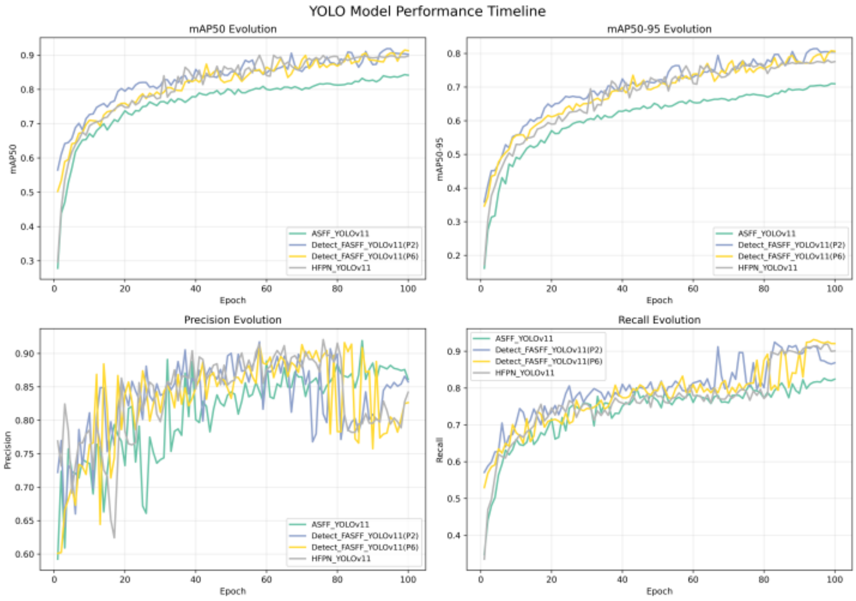

Figure S10. Comparison of mAP50, mAP50-95, recall and precision metrics.

A comparative analysis of the loss function among the two enhanced models and the baseline YOLOv11.

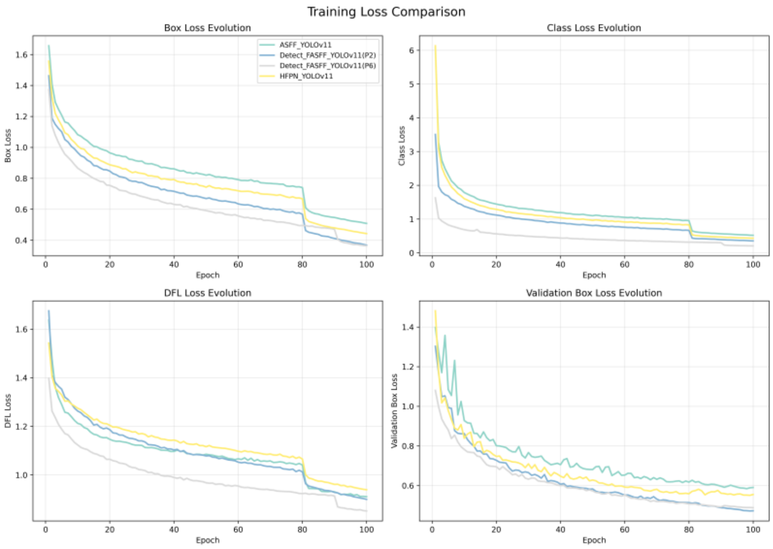

Figure S11. Comparison of loss functions.

The model was optimized using a composite loss function, specifically designed to balance precise spatial localization with accurate behavioral classification, a requirement critical for reliable automated monitoring. This function integrates three components:

Bounding Box Regression Loss (box): Combines Distribution Focal Loss (DFL) and Complete Intersection over Union (CIoU). DFL enhances localization, particularly for small targets, by modeling the probability distribution of bounding box coordinates. CIoU provides a superior geometric penalty by incorporating overlap area, centerfold distance, and aspect ratio consistency.

Classification Loss (ls): Implemented via Binary Cross Entropy (BCE) with label smoothing. It penalizes classification across the defined behavioral states (e.g., 'standing', 'walking', 'foraging'), while label smoothing regularizes the model by preventing overconfident predictions.

Distribution Focal Loss (fl): A dedicated component within L_box that focuses on refining the precision of the predicted coordinate distributions.

Statistical Evaluation Protocol

To ensure the reported performance metrics are robust and not attributable to random training variability, all results are derived from three independent training runs initialized with distinct random seeds (42, 3407, 2026).

Model stability and result variability are quantified using 95% confidence intervals, calculated via the t-distribution to account for the limited number of runs. These intervals are visually presented as error bars in bar charts and as confidence bands in training curves (**Figure S12**). This rigorous approach confirms that the observed performance improvements are statistically reliable.

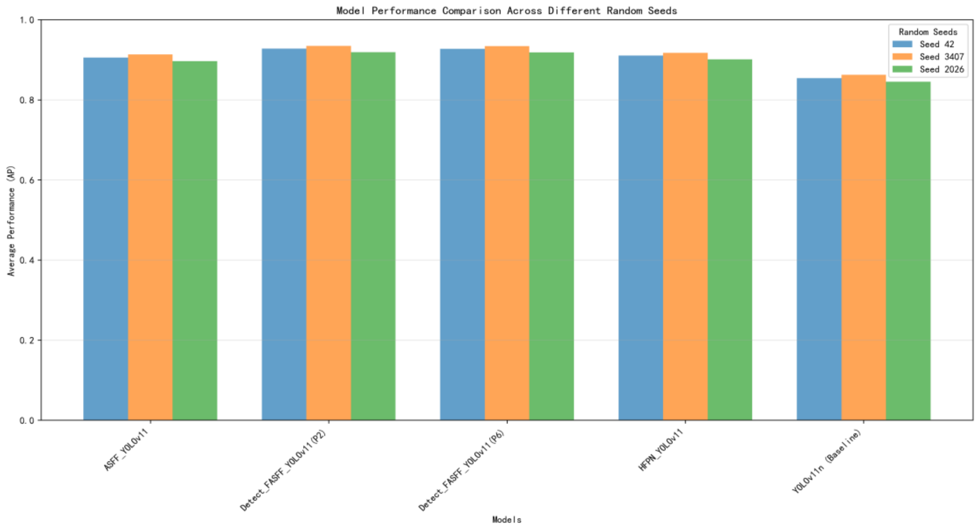

Figure S12. Confidence intervals using 423,407 and 2026 random seeds. Green represents 2,026; blue represents 42; and orange represents 3,407.

To improve the visualization of the model, we presented detailed behavior analysis diagrams and model comparison diagrams, as depicted in **S13**.

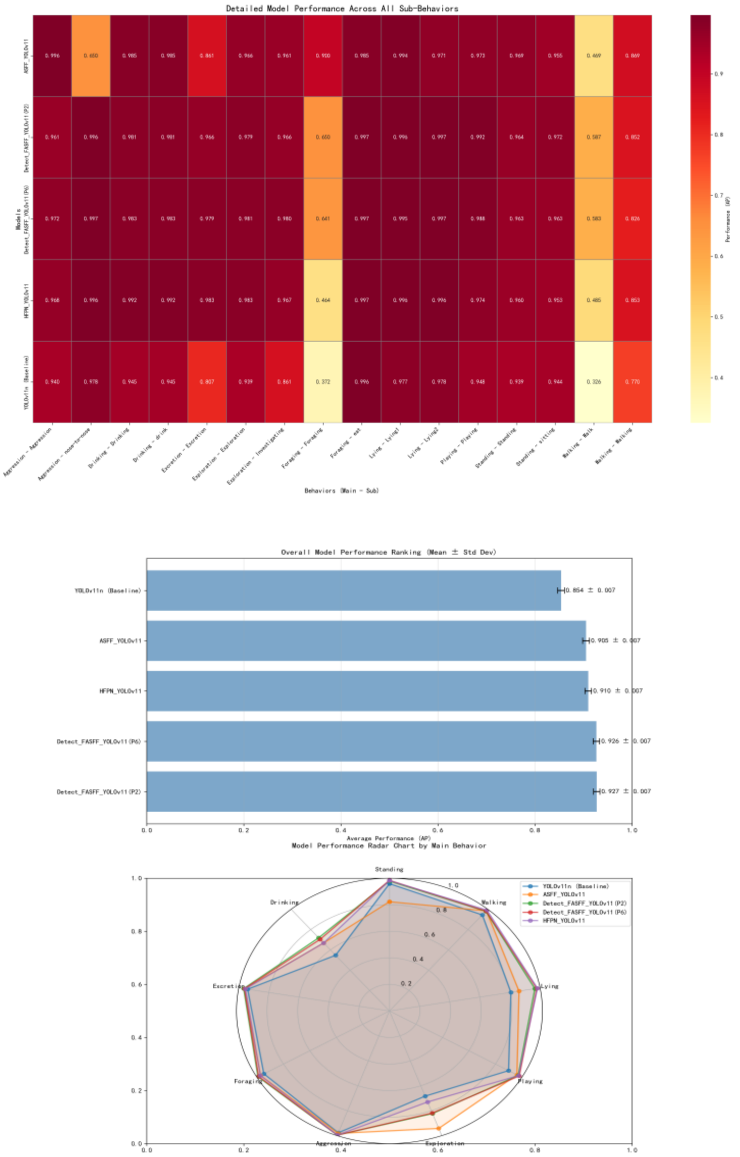

Figure S13. Comparison between the Fine Sub-behavior Analysis Diagram and the Model Diagram.

The enhanced YOLOv11 model was juxtaposed with well-established models such as YOLOv5n, YOLOv7n, YOLOv8n, YOLOv10n, YOLOv12n, YOLOv13n, Faster R-CNN, and RT-DETR. A comprehensive multivariate comparative analysis thoroughly substantiated the remarkable merits of the proposed improvement strategy in intelligent pig health monitoring. The outcomes are presented in **Table S3** below.

Table S3. Table of model evaluation indicators.

Model	mAP_{50} (%)	mAP_{50-95} (%)	FLOPs (G)	Recall (%)	Box (p)	Params (M)	Latency (ms)	FPS (frames per second)
Proposed Models								
ASFF_YOLOv11	0.843	0.71	9.6	0.821	0.878	5.9	7.8	128.8
Detect_FASFF_YOLOv11(P2)	0.919	0.815	8.4	0.911	0.848	4.05	6.5	153.8
Detect_FASFF_YOLOv11(P6)	0.915	0.808	8.1	0.921	0.825	3.9	6.4	156.3
HFPN_YOLOv11	0.895	0.781	13.5	0.919	0.791	11.5	9.4	106.4
Baseline Models								
YOLOv5n	0.51	0.414	25.9	0.502	0.632	16.1	20.4	49.1
YOLOv7n	0.628	0.493	24.8	0.509	0.659	12.8	16.7	59.3
YOLOv8n	0.686	0.536	23.7	0.513	0.702	11.7	12.1	82.6
YOLOv10n	0.743	0.547	22.6	0.538	0.774	10.5	10.6	94.3
YOLOv11n	0.758	0.629	21.3	0.729	0.804	9.4	9.41	104.7
YOLOv13n	0.782	0.658	13.1	0.719	0.828	6.5	8.8	113.6
Faster R_CNN	0.776	0.67	169.7	0.766	0.809	52.8	46.8	21.5
Rt_Detr	0.748	0.636	103.4	0.723	0.752	31.9	35.5	28.2

To conduct a comprehensive evaluation of the model's performance across diverse health states, we present the category average precision (AP) of each architecture in **Table S4**. This analysis enables the identification of the strengths and weaknesses of each category.

Table S4. Comparison of Average Precision (AP) among different improved models and the baseline model.

Model	Standing<br>Standing<br>sitting	Walking<br>Walk<br>run	Lying<br>Lying<br>sleep	Playing<br>Playwright	Exploration<br>Investigating<br>nose-to-nose	Aggression<br>fight	Foraging<br>Eat<br>nose-poke-elsewhere	Excretion<br>other	Drinking<br>drink
YOLOv11n<br>(Baseline)	0.934<br>0.939	0.765<br>0.321	0.972<br>0.973	0.943	0.934<br>0.856	0.935	0.973<br>0.367	0.802	0.940
ASFF_YOLOv11	0.964<br>0.950	0.864<br>0.464	0.990	0.966	0.968<br>0.961	0.956	0.994<br>0.645	0.895	0.980
Detect_FASFF_<br>YOLOv11(P2)	0.959<br>0.967	0.847<br>0.582	0.993<br>0.995	0.988	0.974<br>0.961	0.956	0.994<br>0.645	0.995	0.976
Detect_FASFF_<br>YOLOv11(P6)	0.958<br>0.958	0.821<br>0.578	0.992<br>0.995	0.983	0.976<br>0.975	0.967	0.995<br>0.636	0.995	0.978
HFPN_YOLOv11	0.955<br>0.948	0.848<br>0.480	0.993<br>0.994	0.969	0.978<br>0.962	0.963	0.994<br>0.459	0.995	0.987

Comparative Analysis of Enhanced YOLO Architectures

All enhanced models incorporating advanced feature fusion modules ASFF_YOLOv11, Detect_FASFF_YOLOv11 and HFPN_YOLOv11 demonstrated significantly superior performance to the baseline YOLOv11n. This finding confirms that optimizing multi-scale feature fusion is essential for improving behavioral recognition accuracy within complex farming environments.

1. ASFF_YOLOv11: A Robust General Enhancer

As detailed in **Table S2**, the ASFF model provided substantial and consistent precision gains across all behavioral categories. It achieved very high scores for major states such as foraging (eat) (0.994) and lying (0.990), validating its core mechanism of deceptively fusing feature pyramid levels via learned spatial weights to mitigate macro-scale conflicts.

However, its performance, while improved, did not reach the optimum for tasks requiring fine-grained discrimination, such as distinguishing sitting (0.950) from standing (0.964) or recognizing the challenging run behavior (0.464). This limitation suggests that spatial weighting alone may be insufficient to fully exploit discriminate micro features. Furthermore, the model lacks an explicit design to compensate for the loss of shallow, detailed information in deeper layers (i.e., cross-scale context), which likely constrains its upper performance for detail-dependent behaviors like nose-poke-elsewhere (0.645).

2. Detect_FASFF_YOLOv11: A Flexible Specialist Optimizer

The Detect_FASFF_YOLOv11 model, enabling flexible feature enhancement at the detection head, achieved peak performance on selected feature scales. For instance,

the FASFF(P2) variant attained near perfect precision for lying (sleep) and excretion (both

0.995), while FASFF(P6) excelled at exploration (nose-to-nose) and aggression. This demonstrates a capacity for highly optimized representation within specific scale ranges, allowing customization for particular behavior types.

A primary disadvantage is the strong dependency of optimal performance on manually selected feature levels, with P2 and P6 each excelling at different tasks (e.g., P2 for walk at 0.847 vs. P6 for run at 0.578). This leads to performance instability and necessitates extensive tuning. Generalization can also be uneven across scales, as evidenced by FASFF(P6)'s performance on walk (0.821) being slightly lower than that of ASFF (0.864).

3. HFPN_YOLOv11: A Systematic Balancer for Application Objectives

The HFPN_YOLOv11 model showed leading performance in recognizing key welfare-relevant behaviors. It achieved the best scores for exploring (investigating: 0.978), walking (walk: 0.848) and drinking (0.987), validating the efficacy of its dual-path attention and cross-scale fusion in capturing fine-grained, dynamic motions.

Notably, HFPN was the only model that ranked within the top two across all nine major behavioral categories. Its performance gaps were minimal (within 0.02 of the best model) for most categories, including standing, lying, and foraging (eat). This indicates that its sequential fusion pipeline generates a unified, highly discriminate, and generalization feature representation.

The model also clearly delineates future challenges. Its suboptimal scores on run (0.480) and nose-poke-elsewhere (0.459) highlight the current limitations of a purely visual, single-frame attention mechanism against extreme motion blur and background similarity. The

architectural complexity of HFPN introduces a slight computational overhead compared to ASFF, suggesting a potential need for lightweight refinement in edge deployment scenarios.

Synthesis

In summary, the three models embody distinct feature fusion philosophies: ASFF_YOLOv11 serves as a robust general enhancer, Detect_FASFF_YOLOv11 acts as a flexible specialist optimizer, and HFPN_YOLOv11 functions as an application-targeted systematic balance. This comparative analysis elucidates the performance trade-offs associated with different architectural strategies for automated animal behavior monitoring.
